# Supplementary material for: Repeatability of wildlife surveys for estimating abundance: A method to assess the consistency of detection probability and animal availability
Source: PLoS One. 2025 Apr 23;20(4):e0321619. doi: 10.1371/journal.pone.0321619 (PMC12017516; doi:10.1371/journal.pone.0321619)
Supplement: S1 Appendix — (DOCX) [file pone.0321619.s001.docx]

**S1 Appendix.**

To attempt to better explain variation in detection probability among sites and years, we extracted a green-up index (Normalized Difference Vegetation Index, NDVI) from satellite imagery from NOAA (NOAA CDR Program. NOAA Climate Data Record (CDR) of VIIRS Normalized Difference Vegetation Index (NDVI), Version 1. NOAA National Centers for Environmental Information. https://doi.org/10.25921/gakh-st76.). We developed a framework to process this data, which resulted in weekly temporal resolution and 4,300 m spatial resolution.

Cloud cover prevents satellites from capturing a meaningful measure of NDVI when present, and this issue is detectable as NDVI values close to zero following prior higher values. To navigate this, we downloaded imagery for three consecutive days each week for the length of sampling dates each year. We extracted NDVI data from images within ArcGIS Pro using the *Make NetCDF Raster Layer* tool. We extracted this data to R and calculated the mean NDVI value in each county, each day. We specified that mean NDVI could not decrease in a county over the course of a Spring. When a quality sample was not taken within than 7 days prior to the observation date, we interpolated NDVI with a straight line using the two nearest quality samples. We joined this cleaned NDVI data to our spotlight survey dataset by the nearest date a measurement existed (maximum = 3 days from survey date).
