# Supplementary material for: Repeatability of wildlife surveys for estimating abundance: A method to assess the consistency of detection probability and animal availability
Source: PLoS One. 2025 Apr 23;20(4):e0321619. doi: 10.1371/journal.pone.0321619 (PMC12017516; doi:10.1371/journal.pone.0321619)
Supplement: S1 Table — Only an intercept was fit on abundance. Models ranked by ΔAIC. (DOCX) [file pone.0321619.s002.docx]

**S1 Table**.

|  |  | **Predictor variables** | | | | |  |  |  |
| --- | --- | --- | --- | --- | --- | --- | --- | --- | --- |
| **p intercept** | **γ intercept** | **day of year** | **humidity** | **temperature** | **year** | **day of year x year** | **df** | **AIC** | **Δ AIC** |
| -0.70 | 5.3 | -0.05 |  |  | + | + | 8 | 2089.4 | 0.0 |
| -0.70 | 5.3 | -0.05 |  | 0.00 | + | + | 9 | 2090.7 | 1.3 |
| -0.70 | 5.3 | -0.05 | 0.00 |  | + | + | 9 | 2091.2 | 1.8 |
| -0.69 | 5.3 | -0.05 | 0.00 | 0.00 | + | + | 10 | 2092.4 | 3.0 |
| -0.66 | 5.3 | -0.04 |  |  | + |  | 6 | 2116.1 | 26.7 |
| -0.66 | 5.3 | -0.04 |  | 0.00 | + |  | 7 | 2117.7 | 28.4 |
| -0.66 | 5.3 | -0.04 | 0.00 |  | + |  | 7 | 2118.0 | 28.6 |
| -0.66 | 5.3 | -0.04 | 0.00 | 0.00 | + |  | 8 | 2119.7 | 30.3 |
| 0.12 | 5.3 | -0.04 |  |  |  |  | 4 | 2384.6 | 295.2 |
| 0.12 | 5.3 | -0.04 |  | 0.00 |  |  | 5 | 2384.8 | 295.4 |
| 0.11 | 5.3 | -0.04 | 0.00 |  |  |  | 5 | 2386.6 | 297.2 |
| 0.12 | 5.3 | -0.04 | 0.00 | 0.00 |  |  | 6 | 2386.7 | 297.4 |
| -0.41 | 5.3 |  | 0.00 | 0.00 | + |  | 7 | 2467.3 | 378.0 |
| -0.41 | 5.3 |  | 0.00 |  | + |  | 6 | 2470.9 | 381.5 |
| -0.38 | 5.3 |  |  | -0.01 | + |  | 6 | 2472.4 | 383.1 |
| -0.39 | 5.3 |  |  |  | + |  | 5 | 2477.1 | 387.7 |
| 0.14 | 5.3 |  | 0.00 | -0.01 |  |  | 5 | 2666.3 | 577.0 |
| 0.13 | 5.3 |  |  | -0.01 |  |  | 4 | 2666.9 | 577.6 |
| 0.13 | 5.3 |  | 0.00 |  |  |  | 4 | 2670.8 | 581.5 |
| 0.13 | 5.3 |  |  |  |  |  | 3 | 2672.4 | 583.0 |
