# Supplementary material for: Repeatability of wildlife surveys for estimating abundance: A method to assess the consistency of detection probability and animal availability
Source: PLoS One. 2025 Apr 23;20(4):e0321619. doi: 10.1371/journal.pone.0321619 (PMC12017516; doi:10.1371/journal.pone.0321619)
Supplement: S2 Table — We selected the best fit model from S1 Table (i.e., null model here) and fit four additional models that each had one site-level covariate. Separate models were fit for each site-level covariate because of correlation among these variables (S5 Fig). Models are ranked by ΔAIC. (DOCX) [file pone.0321619.s003.docx]

**S2 Table**.

| **Additional Predictors** | **AIC** | **Δ AIC** |
| --- | --- | --- |
| elevation variation | 1947.1 | 0.0 |
| hay/pasture (%) | 2061.5 | 114.4 |
| crop (%) | 2074.1 | 127.0 |
| forest (%) | 2083.5 | 136.4 |
| null | 2089.4 | 142.3 |
