# Supplementary material for: Repeatability of wildlife surveys for estimating abundance: A method to assess the consistency of detection probability and animal availability
Source: PLoS One. 2025 Apr 23;20(4):e0321619. doi: 10.1371/journal.pone.0321619 (PMC12017516; doi:10.1371/journal.pone.0321619)
Supplement: S3 Table — We used the top model from S2 Table (i.e., intercept model here) and built nine additional models, each represented by a row. Site-level covariates were fit in separate models because of correlation among these variables (S5 Fig). Models are ranked by ΔAIC. (DOCX) [file pone.0321619.s004.docx]

**S3 Table**.

| **Abundance Predictors** | **AIC** | **Δ AIC** |
| --- | --- | --- |
| hay/pasture (%) | 1933.0 | 0.0 |
| year + hay/pasture (%) | 1936.6 | 3.5 |
| crop (%) | 1936.7 | 3.7 |
| forest (%) | 1940.1 | 7.1 |
| year + crop (%) | 1940.3 | 7.3 |
| year + forest (%) | 1943.7 | 10.7 |
| intercept | 1949.0 | 15.9 |
| elevation variation | 1950.5 | 17.5 |
| year | 1952.3 | 19.3 |
| year + elevation variation | 1954.0 | 20.9 |
