# Supplementary figures and images for: Repeatability of wildlife surveys for estimating abundance: A method to assess the consistency of detection probability and animal availability
Source: PLoS One. 2025 Apr 23;20(4):e0321619. doi: 10.1371/journal.pone.0321619 (PMC12017516; doi:10.1371/journal.pone.0321619)

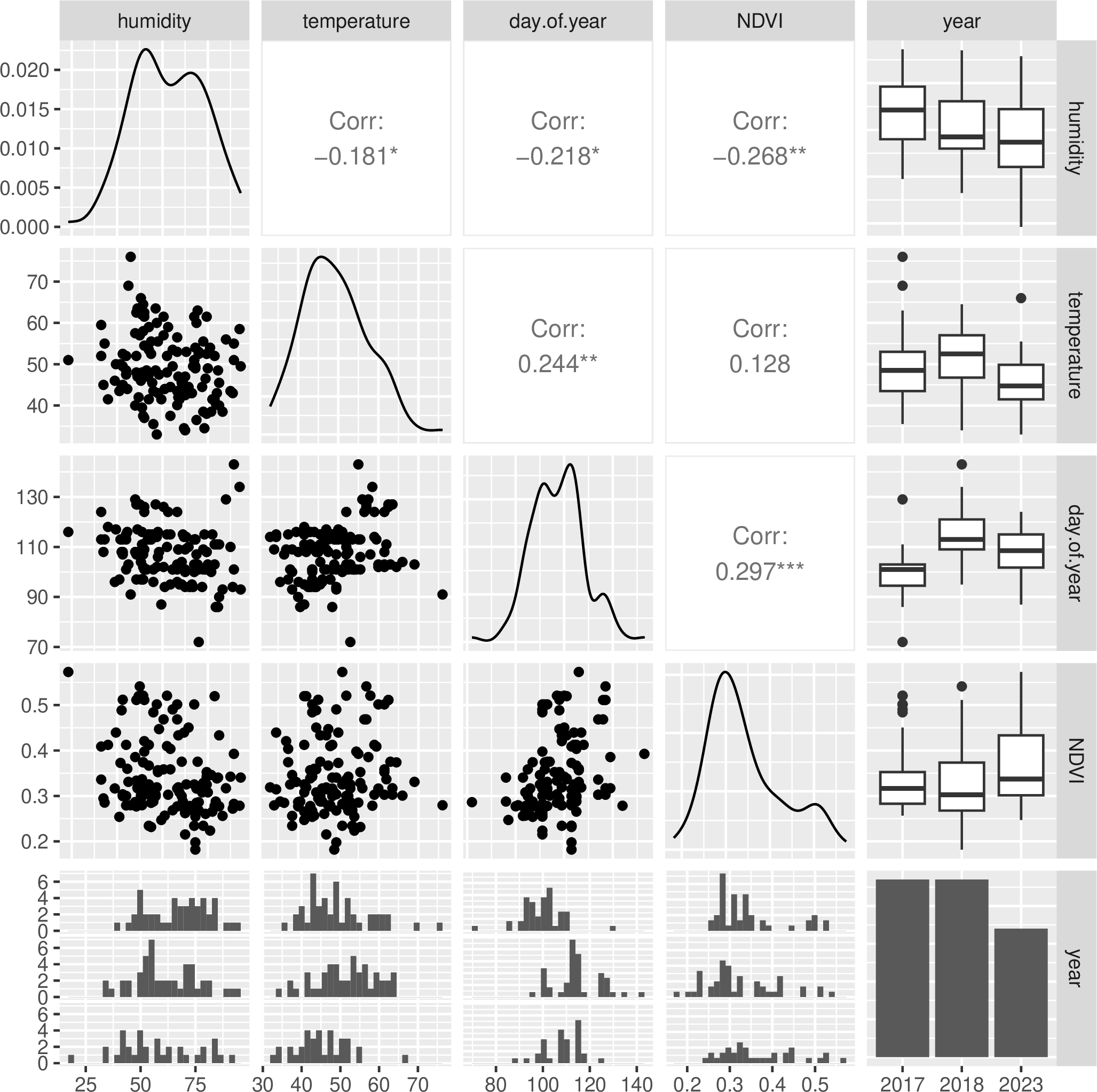

Supplement: S1 Fig — This figure was created in the R package GGally. Panels on the diagonal represent the univariate distribution for each variable. Panels up and right of the diagonal represents correlation coefficients with significance levels denoted with asterisks. Panels low and left of the diagonal represent bivariate scatterplots. Year was fit as a factor in all models and the distribution of each variable among years is shown with histograms (along bottom row) and boxplots (on right column). (TIF) [file pone.0321619.s005.tif]

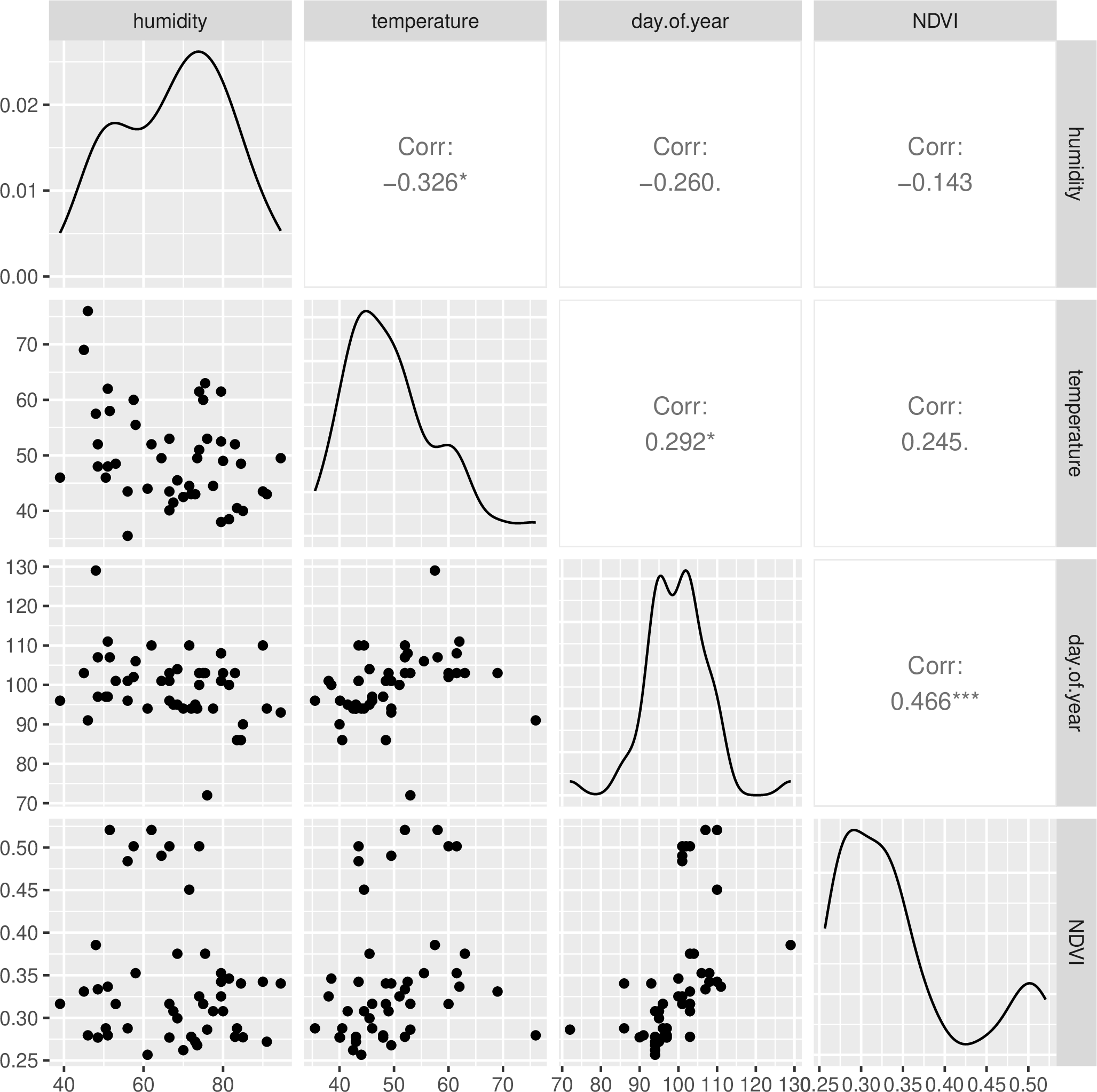

Supplement: S2 Fig — This figure was created in the R package GGally. Panels on the diagonal represent the univariate distribution for each variable. Panels up and right of the diagonal represents correlation coefficients with significance levels denoted with asterisks. Panels low and left of the diagonal represent bivariate scatterplots. (TIF) [file pone.0321619.s006.tif]

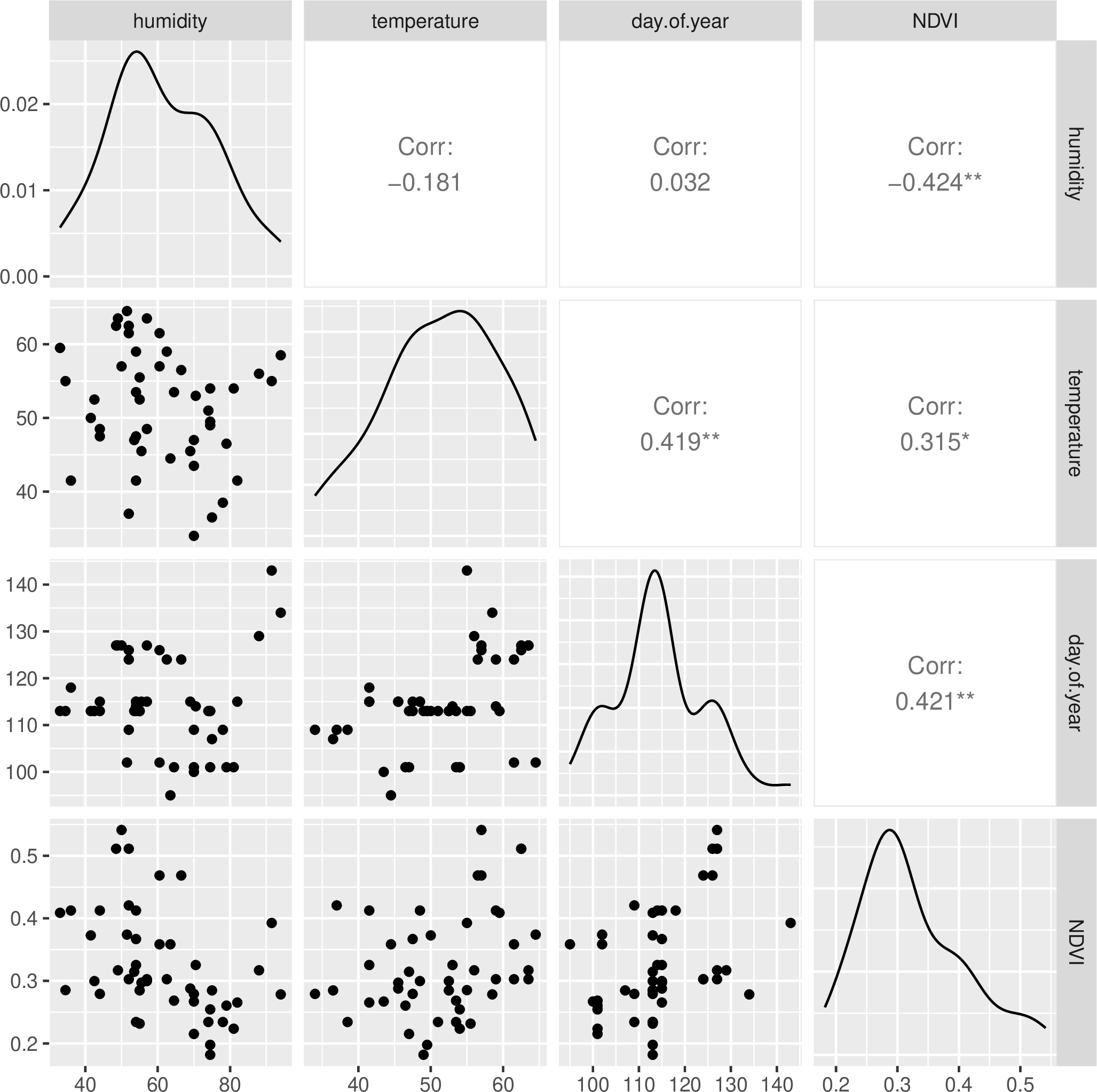

Supplement: S3 Fig — This figure was created in the R package GGally. Panels on the diagonal represent the univariate distribution for each variable. Panels up and right of the diagonal represents correlation coefficients with significance levels denoted with asterisks. Panels low and left of the diagonal represent bivariate scatterplots. (TIF) [file pone.0321619.s007.tif]

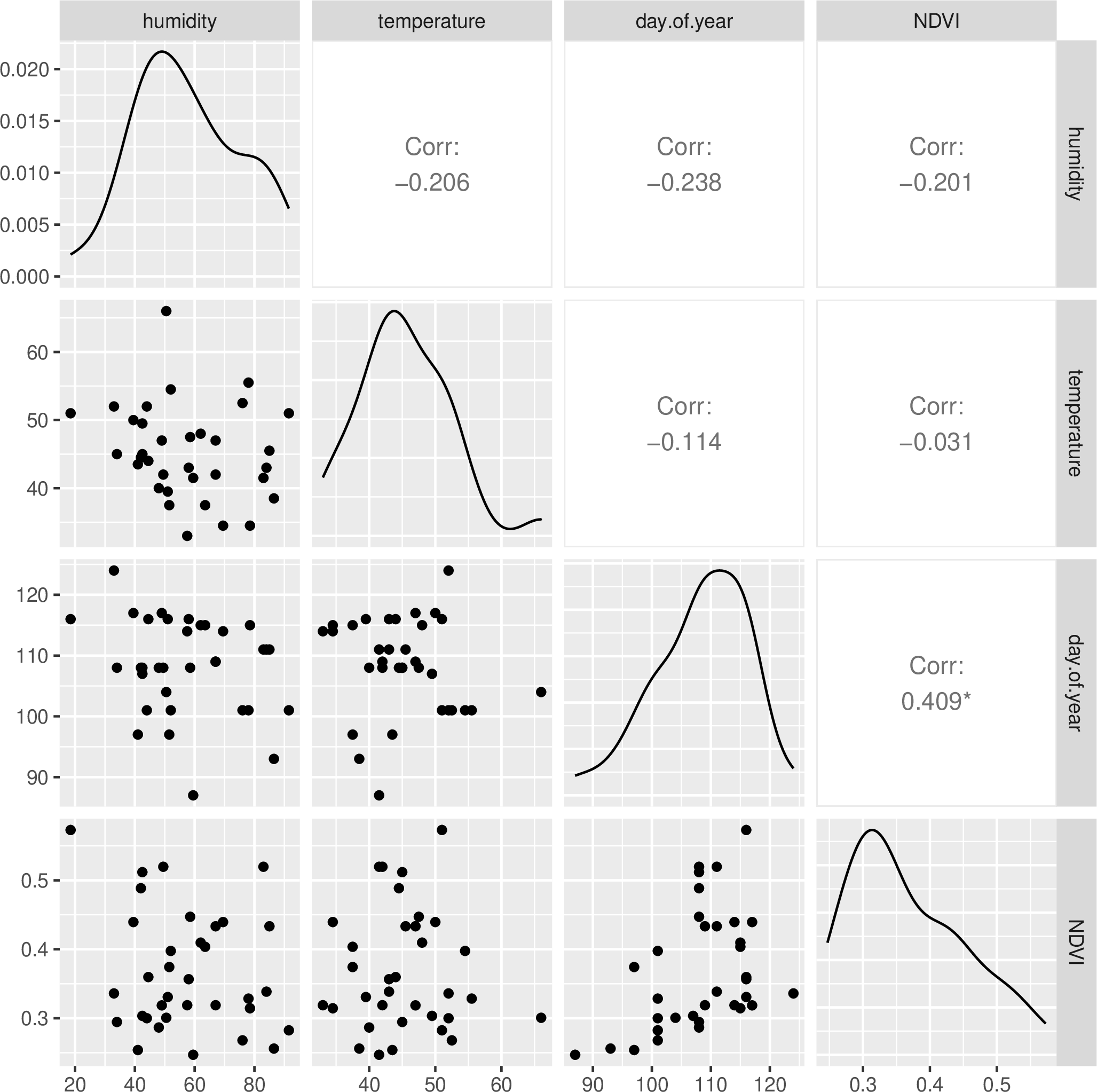

Supplement: S4 Fig — This figure was created in the R package GGally. Panels on the diagonal represent the univariate distribution for each variable. Panels up and right of the diagonal represents correlation coefficients with significance levels denoted with asterisks. Panels low and left of the diagonal represent bivariate scatterplots. (TIF) [file pone.0321619.s008.tif]

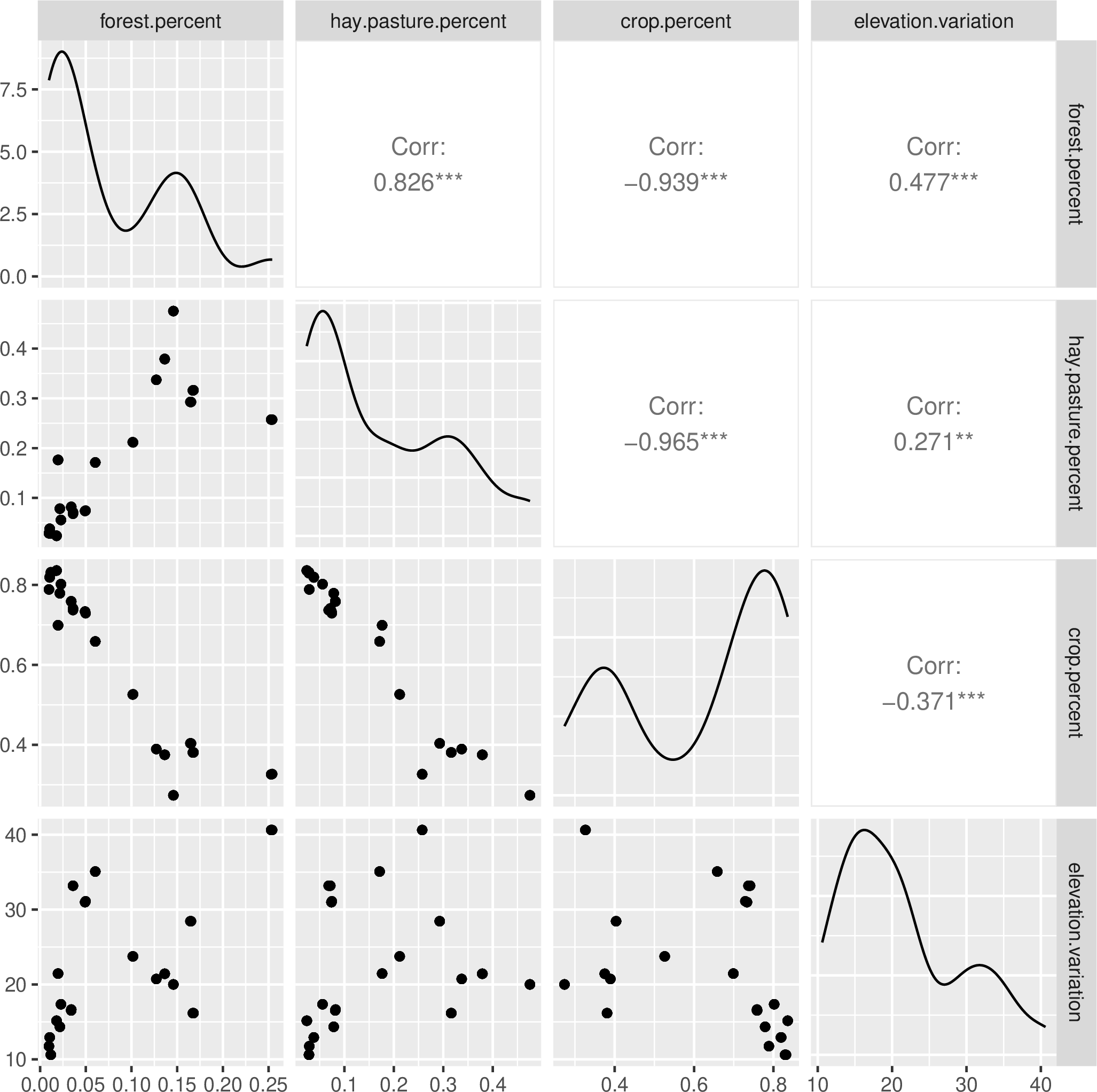

Supplement: S5 Fig — This figure was created in the R package GGally. Panels on the diagonal represent the univariate distribution for each variable. Panels up and right of the diagonal represents correlation coefficients with significance levels denoted with asterisks. Panels low and left of the diagonal represent bivariate scatterplots. (TIF) [file pone.0321619.s009.tif]

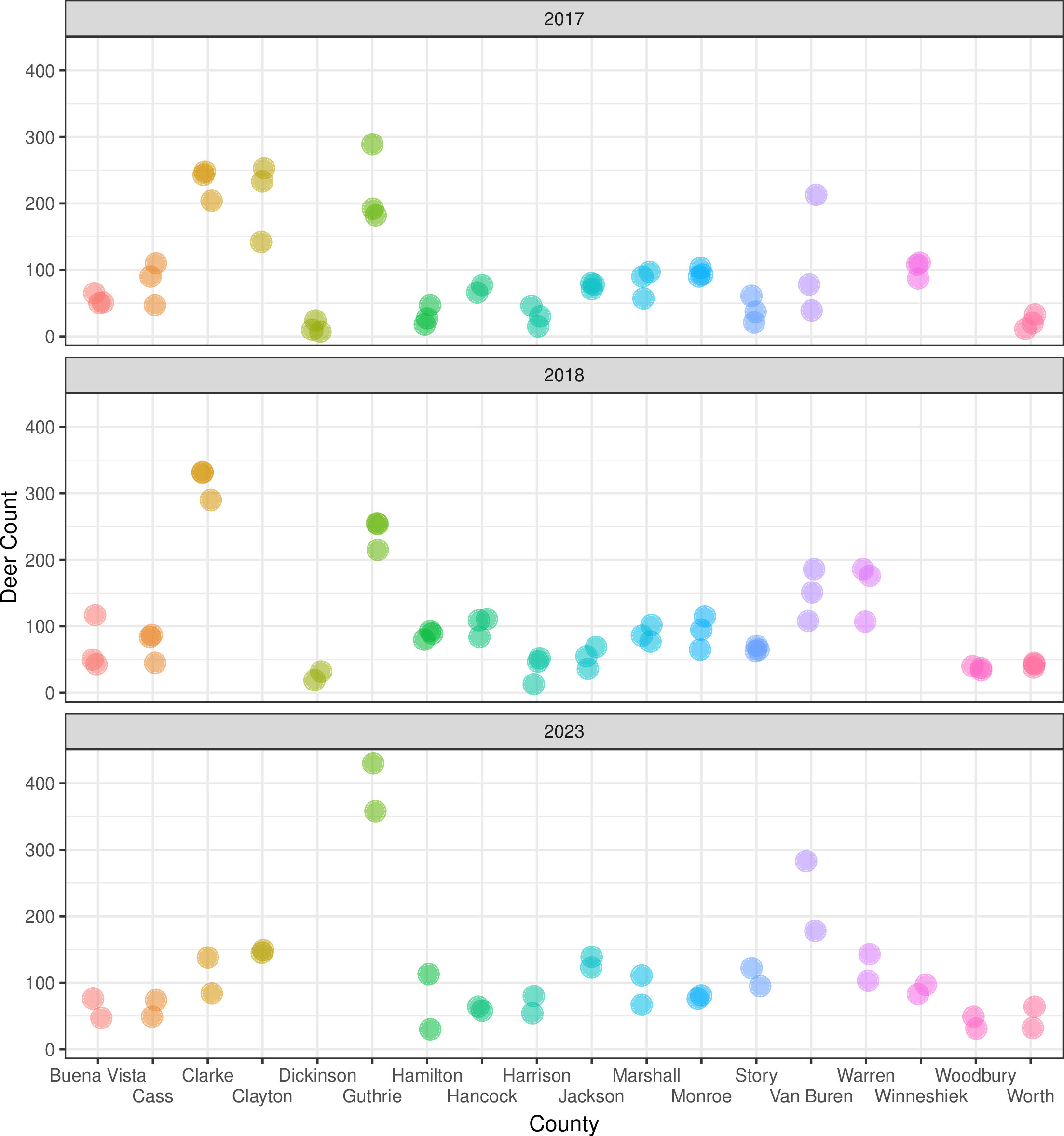

Supplement: S6 Fig — Not all sites were sampled in all years. Each site was temporally replicated three times in 2017 and 2018 and twice in 2023. Points are horizontally jittered to avoid overplotting. (TIF) [file pone.0321619.s010.tif]

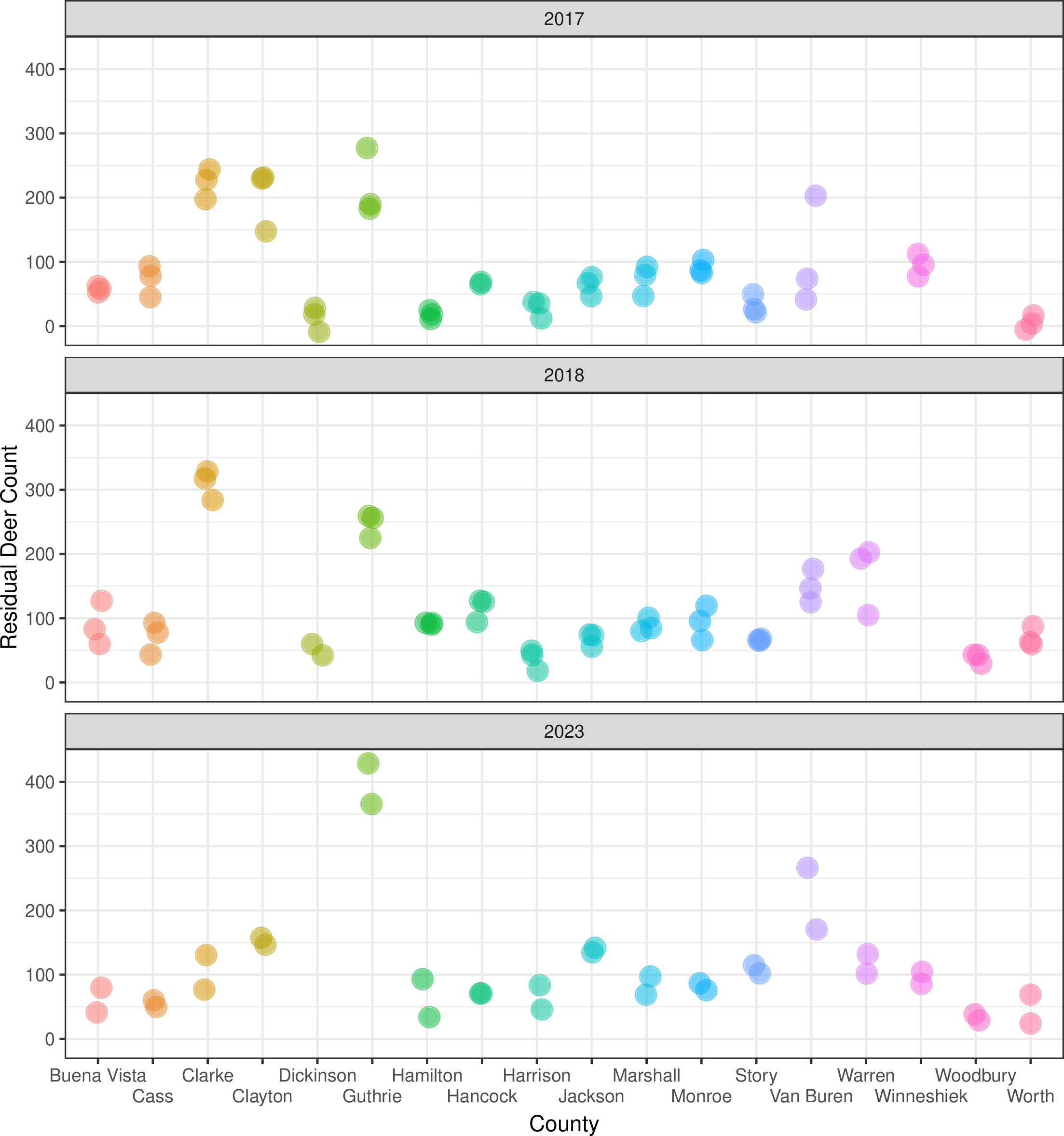

Supplement: S7 Fig — We added the intercept to each point so residual values center around the raw mean. Not all sites were sampled in all years. Each site was temporally replicated three times in 2017 and 2018 and twice in 2023. Points are horizontally jittered to avoid overplotting. (TIF) [file pone.0321619.s011.tif]

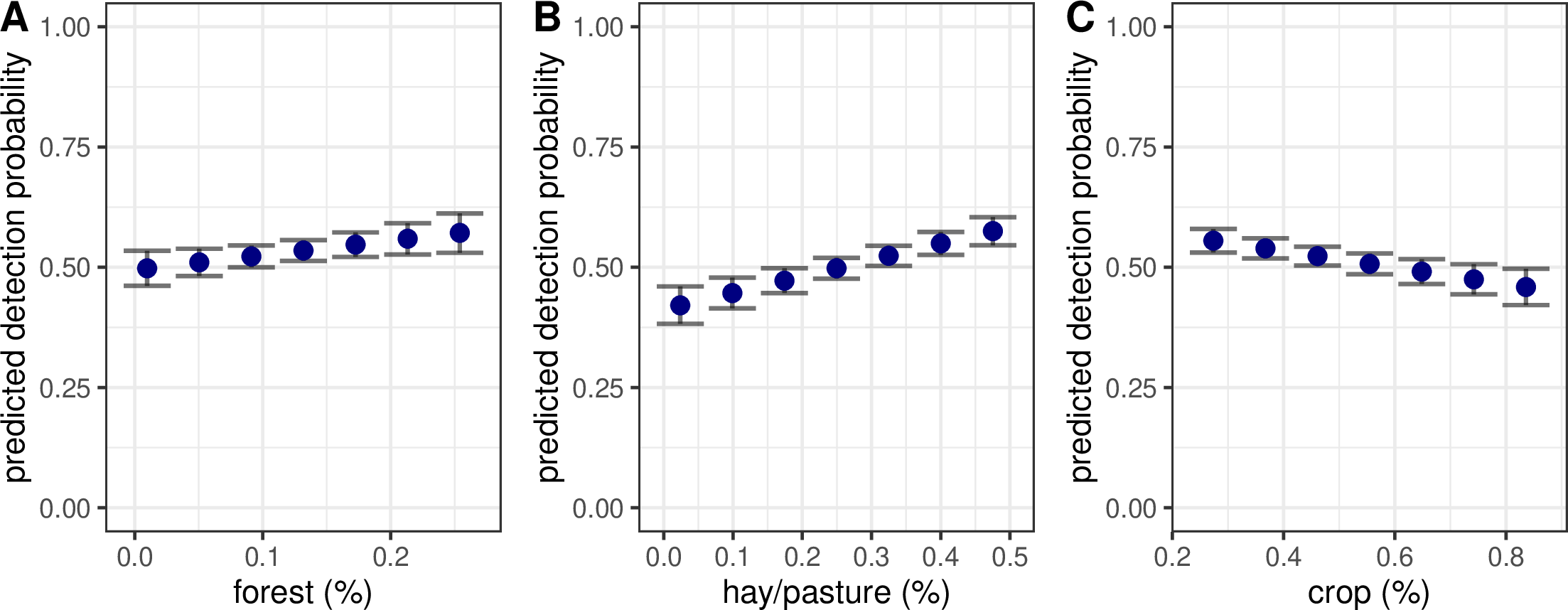

Supplement: S8 Fig — Landcover types were correlated (S5 Fig), and therefore fit in separate models. Points were predicted from the N-mixture model reported in Table 1. Error bars represent ± standard error. (TIF) [file pone.0321619.s012.tif]

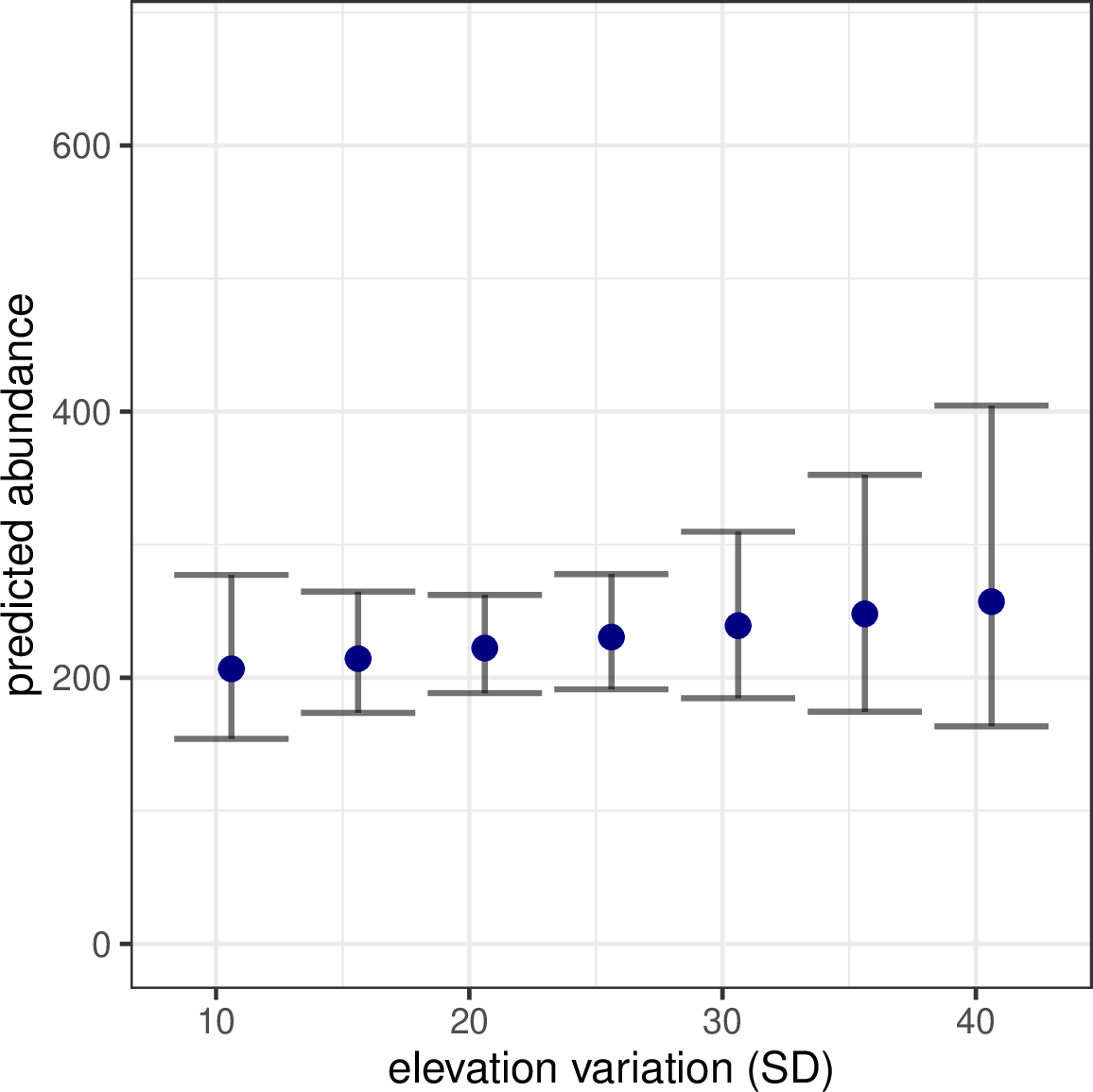

Supplement: S9 Fig — Error bars represent ± standard error. (TIF) [file pone.0321619.s013.tif]
