# Supplementary material for: Repeatability of wildlife surveys for estimating abundance: A method to assess the consistency of detection probability and animal availability
Source: PLoS One. 2025 Apr 23;20(4):e0321619. doi: 10.1371/journal.pone.0321619 (PMC12017516; doi:10.1371/journal.pone.0321619)
Supplement: Attachment — Submitted filename: PONE-D-24-57166_reviewerGM.pdf [file pone.0321619.s015.pdf]

PLOS ONE

Repeatability of spotlight surveys for wildlife abundance estimation

--Manuscript Draft--

**Manuscript Number:**	PONE-D-24-57166
**Article Type:**	Research Article
**Full Title:**	Repeatability of spotlight surveys for wildlife abundance estimation
**Short Title:**	Repeatability of spotlight surveys for wildlife abundance estimation
**Corresponding Author:**	David Delaney<br>Iowa State University<br>Ames, IA UNITED STATES OF AMERICA
**Keywords:**	abundance; availability; density; detection probability; NDVI; spotlight; survey; white-tailed deer; deer
**Abstract:**	A primary consideration of abundance studies that use unmarked animals is whether survey counts accurately reflect the population size or if unknown variation in animal movement or detection probability biases counts irrespective of population size. We posited that high repeatability in counts among temporally replicated surveys would indicate that counts are a good index of abundance. We temporally replicated 49 nocturnal spotlight surveys of white-tailed deer (<i>Odocoileus virginianus</i>) up to three times each (n = 128 total samples) to test the repeatability of this commonly used wildlife monitoring technique. Repeatability was high (r = 0.86), suggesting spring spotlight surveys provide a reliable index of deer population size in Iowa, USA. Fourteen percent of the variation among replicated counts was explained by day of year and, to a lesser degree, a vegetation green-up index. Detection probability was high (~0.70) early in the sampling season and declined considerably during the following 6 weeks. Our findings suggest deer managers should sample prior to green-up in the spring to maximize the proportion of the population that is detectable, and that accounting for seasonality on detection estimation is important for reliable abundance estimates if sampling occurs over a range of phenological progression. Finally, we show that temporal replication of surveys is a logistically feasible method to assess the reliability of abundance estimates from single-visit sampling designs.
**Order of Authors:**	David Delaney
	Tyler Harms
	Jonathan Harris
	Dan Kaminski
	Jace Elliott
	Stephen Dinsmore
**Additional Information:**	
**Question**	**Response**
**Financial Disclosure**<br><br>Enter a financial disclosure statement that describes the sources of funding for the work included in this submission. Review the submission guidelines for detailed requirements. View published research articles from *PLOS ONE* for specific examples.<br><br>This statement is required for submission	The author(s) received no specific funding for this work.

Powered by Editorial Manager® and ProduXion Manager® from Aries Systems Corporation

and **will appear in the published article** if the submission is accepted. Please make sure it is accurate.<br><br>**Funded studies**<br>Enter a statement with the following details:<br>• Initials of the authors who received each award<br>• Grant numbers awarded to each author<br>• The full name of each funder<br>• URL of each funder website<br>• Did the sponsors or funders play any role in the study design, data collection and analysis, decision to publish, or preparation of the manuscript?<br><br>Did you receive funding for this work?	
**Competing Interests**<br><br>Use the instructions below to enter a competing interest statement for this submission. On behalf of all authors, disclose any competing interests that could be perceived to bias this work—acknowledging all financial support and any other relevant financial or non-financial competing interests.<br><br>This statement is required for submission and **will appear in the published article** if the submission is accepted. Please make sure it is accurate and that any funding sources listed in your Funding Information later in the submission form are also declared in your Financial Disclosure statement.<br><br>View published research articles from *PLOS ONE* for specific examples.	The authors have declared that no competing interests exist.

**NO authors have competing interests**<br><br>Enter: *The authors have declared that no competing interests exist.*<br><br>**Authors with competing interests**<br><br>Enter competing interest details beginning with this statement:<br><br>*I have read the journal's policy and the authors of this manuscript have the following competing interests: [insert competing interests here]*<br><br>* typeset	
**Ethics Statement**<br><br>Enter an ethics statement for this submission. This statement is required if the study involved:<br><br>• Human participants<br>• Human specimens or tissue<br>• Vertebrate animals or cephalopods<br>• Vertebrate embryos or tissues<br>• Field research<br><br>Write "N/A" if the submission does not require an ethics statement.<br><br>General guidance is provided below. Consult the submission guidelines for detailed instructions. **Make sure that all information entered here is included in the Methods section of the manuscript.**	We collected data by observing animals from a distance, and animals were not captured or marked. Therefore, our study did not require Institutional Animal Care and Use Committee approval.

Powered by Editorial Manager® and ProduXion Manager® from Aries Systems Corporation

**Format for specific study types**<br><br>**Human Subject Research (involving human participants and/or tissue)**<br>• Give the name of the institutional review board or ethics committee that approved the study<br>• Include the approval number and/or a statement indicating approval of this research<br>• Indicate the form of consent obtained (written/oral) or the reason that consent was not obtained (e.g. the data were analyzed anonymously)<br><br>**Animal Research (involving vertebrate animals, embryos or tissues)**<br>• Provide the name of the Institutional Animal Care and Use Committee (IACUC) or other relevant ethics board that reviewed the study protocol, and indicate whether they approved this research or granted a formal waiver of ethical approval<br>• Include an approval number if one was obtained<br>• If the study involved *non-human primates*, add *additional details* about animal welfare and steps taken to ameliorate suffering<br>• If anesthesia, euthanasia, or any kind of animal sacrifice is part of the study, include briefly which substances and/or methods were applied<br><br>**Field Research**<br><br>Include the following details if this study involves the collection of plant, animal, or other materials from a natural setting:<br>• Field permit number<br>• Name of the institution or relevant body that granted permission	
**Data Availability**<br><br>Authors are required to make all data underlying the findings described fully available, without restriction, and from the time of publication. PLOS allows rare exceptions to address legal and ethical concerns. See the PLOS Data Policy and FAQ for detailed information.	Yes - all data are fully available without restriction

Powered by Editorial Manager® and ProduXion Manager® from Aries Systems Corporation

A Data Availability Statement describing where the data can be found is required at submission. Your answers to this question constitute the Data Availability Statement and **will be published in the article**, if accepted.<br><br>**Important:** Stating 'data available on request from the author' is not sufficient. If your data are only available upon request, select 'No' for the first question and explain your exceptional situation in the text box.<br><br>Do the authors confirm that all data underlying the findings described in their manuscript are fully available without restriction?	
**Describe where the data may be found in full sentences. If you are copying our sample text, replace any instances of XXX with the appropriate details.**<br><br>• If the data are **held or will be held in a public repository**, include URLs, accession numbers or DOIs. If this information will only be available after acceptance, indicate this by ticking the box below. For example: *All XXX files are available from the XXX database (accession number(s) XXX, XXX.).*<br>• If the data are all contained **within the manuscript and/or Supporting Information files**, enter the following: *All relevant data are within the manuscript and its Supporting Information files.*<br>• If neither of these applies but you are able to provide **details of access elsewhere**, with or without limitations, please do so. For example:<br><br>*Data cannot be shared publicly because of [XXX]. Data are available from the XXX Institutional Data Access / Ethics Committee (contact via XXX) for researchers who meet the criteria for access to confidential data.*<br><br>*The data underlying the results presented in the study are available from (include the name of the third party*	All relevant data are within the manuscript and its Supporting Information files.

and contact information or URL).<br>• This text is appropriate if the data are owned by a third party and authors do not have permission to share the data.<br><br>* typeset	
Additional data availability information:	

Powered by Editorial Manager® and ProduXion Manager® from Aries Systems Corporation

Manuscript Click here to access/download;Manuscript;Main text.docx

Repeatability of spotlight surveys for wildlife abundance estimation

David M. Delaney[1], Tyler M. Harms[2], Jonathan P. Harris[1], Dan J. Kaminski[2], Jace R. Elliott[2], Stephen J. Dinsmore[1]

[1]Department of Natural Resource Ecology and Management, Iowa State University, Ames, Iowa, USA

[2]Iowa Department of Natural Resources, Boone Research Station, Boone, Iowa, USA

Abstract

A primary consideration of abundance studies that use unmarked animals is whether survey counts accurately reflect the population size or if unknown variation in animal movement or detection probability biases counts irrespective of population size. We posited that high repeatability in counts among temporally replicated surveys would indicate that counts are a good index of abundance. We temporally replicated 49 nocturnal spotlight surveys of white-tailed deer (*Odocoileus virginianus*) up to three times each (n = 128 total samples) to test the repeatability of this commonly used wildlife monitoring technique. Repeatability was high (r = 0.86), suggesting spring spotlight surveys provide a reliable index of deer population size in Iowa, USA. Fourteen percent of the variation among replicated counts was explained by day of year and, to a lesser degree, a vegetation green-up index. Detection probability was high (~0.70) early in the sampling season and declined considerably during the following 6 weeks. Our findings suggest deer managers should sample prior to green-up in the spring to maximize the proportion of the population that is detectable, and that accounting for seasonality on detection

estimation is important for reliable abundance estimates if sampling occurs over a range of phenological progression. Finally, we show that temporal replication of surveys is a logistically feasible method to assess the reliability of abundance estimates from single-visit sampling designs.

Keywords: abundance, availability, density, detection probability, NDVI, spotlight, survey, white-tailed deer

Introduction

Abundance is an important parameter of interest to ecologists and natural resource managers. Estimates of abundance are used to set harvest quotas, assess conservation practices, and test ecological theory [1]. Techniques to estimate abundance from unmarked animals are useful because they require less effort than mark-recapture techniques [2], and can therefore be implemented over a larger area per unit time [3,4]. Line and point transects are commonly implemented techniques to count animals [5,6], which can be paired with N-mixture models [7,8] or distance sampling [9-11] to estimate abundance when detection probability is imperfect. In lieu of estimating detection probability, the number of animals observed per unit effort can be used as an index of relative abundance [4,12]. For all of these techniques, meaningful estimates of abundance require that a survey count be representative of the population at the spatial and temporal extent of the estimate. In particular, variation in either animal density or detectability across space or time can affect the number of animals counted irrespective of population size [13-16].

The reliability of survey counts depends on the homogeneity or predictability of two main components that determine how many animals are counted: (1) availability of animals to sample, and (2) probability of detection given availability (*sensu* [14]). N-mixture and distance sampling models both estimate the abundance of the population that is available to sample, and abundance will be underestimated by the proportion of the population that is unavailable (e.g., availability bias, [17-19]). Availability bias can be reduced by conducting surveys when animals are maximally exposed (e.g., early morning point counts for passerines, [20]) or mathematically accounted for, if quantified, to improve the accuracy of abundance estimates [17,21]. Additionally, N-mixture and distance sampling models estimate the probability of detection of the available animals, which may be affected by a variety of factors including observer [22], weather [23], or land cover [23] and cause variation in counts. If availability and detectability are homogeneous over space and time, temporally replicated surveys should produce similar counts and be ~~deemed~~ a reliable index of the population (e.g., [24]). Confidence in survey counts can also be gained by identifying and modeling factors that explain variation among replicate counts, which enables accurate estimation of abundance (e.g., [14,25,26]). For systems with repeatable counts and predictable detection probability, researchers can reduce visits to one sample per site and reallocate effort elsewhere.

Nocturnal spotlight surveys are commonly used to estimate an index of population size for a variety of wildlife [27-33], but have been criticized as being unreliable because detection probability can vary unpredictably (reviewed in [34]) and be lower than other methods (e.g., thermal imaging, [28]; aerial surveys, [35]). For species that feed in open habitat at night compared to denser diurnal bedding habitat, conducting nocturnal surveys can increase the proportion of the population that is available to sample, and eyeshine (i.e., light reflection off the

tapetum lucidum) can improve detectability [36-38]. Availability is rarely estimated during spotlight surveys and reports of high variability in detection could be a combination of availability and detection [19]. For example, spotlight surveys of white-tailed deer (*Odocoileus virginianus*) in forested regions report high and unpredictable variation in detection probability [27], which is attributed to variation within and among observers, temporal replicate, and spatial location [31,34]. Despite criticism, ~~similar~~ spotlight surveys have been deemed reliable in estimating relative abundance of red deer (*Cervus elaphus*) in forested regions of Italy [39] and France [25] and white-tailed deer in agricultural landscapes of Iowa, USA [40,41]. Regardless of concerns, spotlight surveys are likely to remain a widely used wildlife monitoring technique because of their balance of cost, labor, and assumed accuracy [42]. Given this, robust assessment of potential bias is recommended to improve the accuracy of abundance estimation and increase the ~~usefulness~~ of survey counts [43].

We were interested in whether nocturnal spotlight survey counts of white-tailed deer in Iowa were repeatable from late March to early May when the Iowa Department of Natural Resources conducts annual population monitoring [40,44]. Staff traditionally drive approximately 78 km of gravel roads in each of Iowa's 99 counties with no temporal replication. Our goal was to temporally replicate surveys and assess whether one sample per county is adequate or whether future efforts should trade off such broad spatial monitoring for increased effort per unit area. We hypothesized that if availability and detection probability were stable during this period of spring, temporal replicates would produce consistent counts, suggesting single surveys would provide adequate population indices. We posited that variation in replicate counts resulting from observation-level covariates could be analytically corrected to produce more accurate estimates of population size. In contrast, poor consistency in counts among

temporal replicates that was not explained by covariates would suggest spotlight surveys do not produce meaningful counts during this period and alternative sampling techniques should be explored.

Study Area

The Iowa Department of Natural Resources conducts nocturnal spotlight surveys of white-tailed deer each spring in each county to estimate an index of population size [40,44,45]. These surveys typically occur between mid-March and mid-May. Early during this time period, food is limited and deer frequently feed in open habitat [46-50] and in larger groups [51]. However, constraints on staff time and local weather conditions often cause some counties to be sampled later when spring green-up has occurred to varying degrees.

Surveys for this study were conducted along gravel roads in 18 counties of Iowa, USA (Fig 1). The landcover within 400 m buffers of county-level transects ranged from 1–25 % forest, 2–48 % hay/pasture, and 27–84 % row crop agriculture (data from U.S. Geological Survey National Land Cover Database (NLCD) [52]). The average quality of deer habitat (quantified with a resource selection function from [40]) within 300 m of transects was highly correlated with the average quality of habitat of the broader county they represent (correlation coefficient = 0.97, unpublished data) which is attributable to high spatial coverage (78 km of transect route per county on average) and consideration of available habitat during route selection. Detailed description of the flora, fauna, and landscape of Iowa, as they pertain to deer abundance, occur in Elliott and Harms [53].

Figure 1. Map of study locations in Iowa, USA. Bolded black lines indicate transect routes for the nocturnal spotlight survey used to sample populations of white-tailed deer (*Odocoileus virginianus*).

Methods

To assess the repeatability of survey counts, we temporally replicated surveys in 18 counties up to three times per year for up to three years (2017, 2018, and 2023). Intervals between samples of the same site within years averaged 7.7 days (range 1–28 days). We assumed the sample populations were closed between temporal replicates because no hunting season or birth occurred during this time. Our dataset therefore consisted of 128 samples of 49 populations we assumed were closed.

Surveys began one hour after sunset and were restricted to nights with temperature > 0° C, wind < 24 km/h, relative humidity > 40%, and no rain or fog. Two observers drove an average of 41 km (± 6 SD, range = 30–59) of gravel roads in each county at less than 32 km/h, each scanning with a spotlight on one side of the road. Observers recorded relative humidity (%), and temperature (°C) at the start and end of each survey. These variables were used later as observation covariates in the analysis. We summed the total number of deer observed each survey.

We used ArcGIS Pro (versions 3.3.0) to quantify the percent of each landcover type within 400 m of the transects driven in each county (2021 NLCD, 30-m resolution [52]). We focused on forest, hay/pasture, and crop landcover as they were the dominate cover types in rural

Iowa. We quantified the variation in elevation within 400 m of each transect by calculating the standard deviation of the elevation of 30 m raster cells from a digital elevation model [54].

Analysis

We analyzed data in the R statistical environment (version 4.2.2). We first examined correlations among observation- and site-level covariates using the package *GGally* [55] to determine if we would separate predictors into multiple models to avoid collinearity.

To quantify the repeatability (*R*) of survey counts, we used linear mixed models in the package *rptR* [56] with site–year as a random effect. We calculated both global repeatability (fraction of total variance explained by site–year) and adjusted repeatability (repeatability after accounting for the fixed effects of temperature, humidity, and day of year, (*sensu* [57]). We modeled the number of deer observed during each survey as a response variable with a Gaussian error distribution.

To quantify whether observation-level variables covaried with detection probability, we built a series of N-mixture models in the *unmarked* package [58]. As is the case for most N-mixture models [19,59,60], we were not able to separate detection from availability, ~~and we discuss the likelihood of each in contributing to our estimation of detection probability~~. We modeled abundance with a negative binomial distribution (c-hat = 1.58) because Poisson (Δ Akaike information criterion (AIC) = 2778) and zero-inflated Poisson (Δ AIC = 2780) yielded poorer model fits for intercepts-only models in an *a priori* analysis. We included temperature, humidity, day of year, and year (as a factor) as predictors of detection probability and fit an intercept on abundance. We also included an interaction of day of year and year because sampling began and concluded at different times each year in response to variation in vegetation

green-up phenology among years. We used the dredge function in the package *MuMIn* [61] to run all possible models that varied in the combination of predictors. We determined the best model as the model with the lowest AIC [62,63] and did not consider models with uninformative parameters [64].

Based on results from the prior model, we also calculated an index of vegetation green-up (Normalized Difference Vegetation Index, NDVI, S Appendix 1). To do this, we used NDVI data available from the National Oceanic and Atmospheric Administration Climate Data Record [65], which was correlated with day of year (r = 0.30 in global dataset; r = 0.41–0.47 within year subsets, Figs S1–4). We built two additional models to test how green-up compared with day of year in predicting detection probability: (1) a model with NDVI and year as predictors and (2) a model with an interaction of NDVI and year as predictors.

To test whether site-level variables covaried with detection probability, we selected the top model and added the following predictors: elevation variation, forest percent, hay/pasture percent, and crop percent. Each additional predictor was fit in a separate model because of high correlation among these variables (Fig S5).

To test whether site-level variables covaried with abundance, we selected the top model for detection probability and ran a series of models including the addition of elevation variation, forest percent, hay/pasture percent, and crop percent as predictors on abundance. Again, each of these predictors was run in separate models because of correlation among these variables. Each model of abundance included transect length as a covariate to account for variable sampling effort among sites. We considered the model with the lowest AIC that included covariates on both detection probability and abundance as our top model.

Results

Repeatability

Survey counts were very repeatable, both globally (R = 0.86 [95% CI = 0.78–0.92]; Fig S6) and after accounting for other potential sources of variation (R = 0.88 [95% CI = 0.81–0.93]; Fig S7). Accounting for observation-level covariates reduced the unexplained variance among temporal replicates (i.e., the within site–year variance) by 14% (from 911 [95% CI = 648–1193] to 788 [CI = 551–1038]).

Detection

Detection probability declined with increasing day of year and the magnitude of this effect varied among years (Table 1; Fig 2A; S Table 1). Detection probability also declined with increasing NDVI and this effect varied among years (Fig 2B). However, the best NDVI model (i.e., NDVI x year) explained considerably less variation than the best day-of-year model (Δ AIC = 107).

Table 1. Results from the best fit N-mixture model assessing the detection probability and abundance of white-tailed (*Odocoileus virginianus*) deer during nocturnal spotlight surveys in Iowa, USA. Coefficients for year are compared to 2017. Hay/pasture percent was highly correlated with forest (r = 0.83) and crop percent (r = -0.97) and each are also significant predictors of abundance when included in place of hay/pasture percent (forest β = 3.9, CI = 1.6–6.2; crop β = -1.5, CI = -2.2– -0.7). Dispersion estimate = 1.5 ± 0.2 SE (z = 7.3, p <0.001).

	Estimate	lower 95% CI	upper 95% CI	P
**Abundance (log-scale):**				
intercept	5.36	5.22	5.50	0.000

transect length	-0.02	-0.04	0.01	0.162
hay/pasture percent	2.32	1.30	3.33	0.000
**Detection (logit-scale)**				
intercept	-1.02	-1.11	-0.92	0.000
year (2018)	1.21	1.07	1.35	0.000
year (2023)	1.09	0.90	1.29	0.000
day of year	-0.05	-0.06	-0.05	0.000
elevation variation	0.06	0.05	0.07	0.000
year (2018):day of year	0.03	0.02	0.03	0.000
year (2023):day of year	0.01	0.00	0.02	0.042

Figure 2. Predicted detection probability of white-tailed deer (*Odocoileus virginianus*) during nocturnal spotlight surveys in Iowa, USA depending on (A) day of year and (B) Normalized Difference Vegetation Index (NDVI). For (A) points were predicted from the N-mixture model reported in Table 1. Day of year and NDVI were correlated from r = 0.41–0.49 within years; we therefore fit (B) NDVI in a separate model in place of day of year for comparison. Error bars represent ± standard error and each year is a different color (dark purple = 2017, teal = 2018, yellow = 2023).

The top model with site-level covariates on detection probability included elevation variation (Table S2) in which sites with greater elevation variation were predicted to have greater detection probability (Table 1; Fig 3). Other site-level covariates explained variation in detection to a lesser degree but were still significant predictors (Fig S8). Forest percent (β = 1.21, 95% confidence interval (CI) = 0.14–2.29) and hay/pasture percent (β = 1.38, CI = 0.88–1.87) positively covaried with detection probability, whereas crop percent had a negative relationship with detection (β = -0.69, CI = -1.03– -0.35).

Figure 3. Predicted detection probability of white-tailed deer (*Odocoileus virginianus*) during nocturnal spotlight surveys in Iowa, USA depending on variation in elevation. Points were predicted from the N-mixture model reported in Table 1. Error bars represent ± standard error.

Abundance

Abundance was best predicted by the percent of hay/pasture landcover, followed by crop percent and forest percent (Table S3). Abundance increased at sites with greater hay/pasture percent (β = 2.32, CI = 1.30–3.33) and forest percent (β = 3.90, CI = 1.62–6.18), and decreased at sites with greater crop percent (β = -1.45, CI = -2.17– -0.73; Fig 4). Elevation variation did not affect abundance (β = 0.01, CI = -0.01–0.03; Fig S9).

Figure 4. Predicted abundance of white-tailed deer (*Odocoileus virginianus*) during nocturnal spotlight surveys in Iowa, USA depending on percent of different landcover classifications. Points were predicted from the N-mixture model reported in Table 1 (B) or its caption (A & C). Error bars represent ± standard error.

Discussion

A common question in wildlife research is whether survey counts of animals are repeatable and provide an accurate index of abundance or whether counts vary due to variation in detectability or availability of animals. We found very high consistency in counts of white-tailed deer among temporal replicates during spring in an agriculturally dominated landscape of the Midwest, USA. We were able to explain additional variation in detection by modeling day of year and elevation variation, which should increase the accuracy of abundance estimates.

Repeatability

We argued that confidence in survey counts could be gained from either (1) low variation in counts among temporal replicates or (2) an ability to explain variation in counts with covariates. Given eighty-six percent of the variation in survey counts was explained by site–year and only 14% of the variation was attributable to differences among temporal replicates, survey counts in our system seemed reliable because of low variation in counts among temporal replicates. Nevertheless, we were also able to explain an additional 14% of the unexplained variation in temporal replicates with observation covariates, increasing the amount of variation explained by site–year to 88% and further increasing our confidence in the usefulness of individual survey counts in our system.

Detection probability

Day of year had the strongest effect on detection probability of any observation-level covariate. Detection probability decreased as day of year increased, which we hypothesized was caused by green-up progression through spring. We were therefore surprised that NDVI explained less variation in counts than day of year. We note that the high correlation of day of year and NDVI, particularly within years, hinders our ability to parse effects between these two variables. Regardless of the mechanism causing variation in counts among temporal replicates, our results suggest white-tailed deer managers should survey early in spring prior to significant green-up to increase the proportion of the population detectable with nocturnal spotlight surveys.

Advancing green-up could reduce detection probability by increasing visual complexity on the landscape, and reduce availability if deer increase the use of forest because of new food in

the form of leaf buds (*sensu* [66]). Like other ungulates, white-tailed deer tend to increase group size in late winter and break into smaller groups as spring advances [51,67-69], which could affect detectability. Group size is probably driven by changes in habitat use [67,68]. Ungulates occur in larger groups in open habitat where group vigilance may confer benefits [67,68,70,71]. As deer move to denser landcover where avoiding predator detection altogether is more feasible, deer shift to smaller groups, probably because the benefits of stealth outweigh the benefits of group vigilance. Food may be more scattered in forested habitat, dispersing deer on the landscape and indirectly reducing group size [67]. Day of year could have additional effects on deer behavior as pregnant females separate from groups in preparation for parturition (May–June in Iowa [72]; see also [51]). Lastly, agriculture fields are increasingly worked during this time period, which may disturb deer from open fields and likely reduces food potential if tilled.

Elevation variation was the only site-level covariate to have a considerable effect on detection probability. Percent forest, hay/pasture, and crops had significant associations with detection probability, but these may result from percent landcover correlations with elevation variation (Fig S5). We hypothesized that elevation variation could influence detection probability by increasing the visual complexity of the landscape and increasing the difficulty for an observer to detect deer. In contrast, we observed higher detection probability at sites with greater elevation variation. Sites with greater elevation variation likely have smaller viewsheds, which reduces the area that is sampleable (*sensu* [37,73]). Because elevation variation is likely to shorten the viewshed closer to the transect where visibility is greater, average detection probability over the area that can be sampled should be higher. Smaller viewsheds may also reduce observer fatigue [74] and focus observers on areas where deer could be detected. Topographically diverse sites have also produced reliable spotlight survey estimates of red deer in mountainous regions of Italy

[39] and France [25], and aerial surveys of mule deer (*Odocoileus hemionus*) found higher detection in areas with increased ruggedness [74].

Abundance

Abundance was higher at sites with a greater proportion of forest and hay/pasture cover and was lower at sites with greater landcover in row crop agriculture. Forests provide bedding cover and woody browse that deer rely on, and the amount of forest has been posited as the main factor limiting population size in the Midwest [75]. Corn and soybeans serve as major food sources during the summer and autumn and offer bedding cover when crops are adequately high. After crops are harvested, deer must be able to seek retreat sites that typically exist in Iowa as various types of forests (e.g., oak and hickory upland, willow and maple riparian areas) or other persistent cover in landscapes void of forests (e.g., marsh wetlands, tallgrass prairies). Thus, while it is possible that deer are negatively affected by row crop agriculture, abundance was probably lower at sites with increasing crop percent because of the correlated lack of cover at these sites.

Comparison with other systems

The question of whether survey counts provide unbiased indices of their population is widespread among wildlife studies [14,15,26,76-78]. Yet, this question is particularly contentious for road-based nocturnal spotlight surveys of white-tailed deer (reviewed in [34]), providing a wealth of study to compare the conditions that determine whether a system can accurately be surveyed with counts of unmarked animals. Why were counts consistent and detection probability predictable in our study but not others? First, we surveyed during a time

period that animals were maximally exposed (i.e., at night during early spring), which led to high detection probability. Population estimates from unmarked populations tend to be less accurate when detection probability is low [79-81]. Studies reported unpredictable variation in detection of deer when sampled in July and August in approximately 93% forested landcover [31,34]. Limited food resources within forest during early spring probably create a stronger pressure on deer to feed in exposed areas than summer. Second, we sampled at a time of year when deer movement and landscape use is relatively stable and sampled only on nights with similar environmental conditions. This gives staff flexibility on when they collect data, which is needed when sampling a large spatial extent, and reduces the complexity of covariate structure needed to represent the data generating process. Third, we sampled sites with ≤25% forest cover. Other studies have suggested forested landcover increases the difficulty in reliably estimating detection because visibility is impeded [27,31,34]. We actually found a slight positive association between percent forest and detection, which may result from smaller viewsheds (discussed above). We suspect studies that find high variation in detection probability in highly forested areas are probably the result of temporal variation in animal availability because more food options exist within forest during summer. In support of this, nocturnal spotlight surveys of red deer in highly forested landcover (80% forest [25]; 73% forest [39]) find high and stable detection probabilities in early spring when quality food is limited to open landcover. Nevertheless, the ecology of deer in a warmer climate with 93% forest cover may simply not be predictable enough to accurately implement spotlight surveys [34]. In general, we suggest counts were repeatable in our system because we surveyed when deer were available to sample, landscape use was stable, and the primary influence of heterogeneity (day of year/green-up) was quantifiable. In other words, timing of surveys allowed us to minimize unmodeled heterogeneity in detection probability.

An important assumption of transect sampling is that transects are randomly located in the area that abundance is estimated [9]. Road based surveys violate this assumption and can be especially problematic if animals or habitat quality are distributed nonrandomly from roads [34,82,83]. Because our goal was to assess the temporal consistency of counts, the nonrandomness of transects was not an issue for our current study. Researchers conducting road- or trail-based surveys should consider the potential consequences of non-random transects if extrapolating density outside of the visible area. Because of their logistic ease, road-based surveys are likely to remain a widely used wildlife monitoring technique.

Methodological considerations for assessing detection probability and availability

There is great interest in validating the reliability and assumptions of abundance estimation techniques for unmarked animals [24,43,76,78,84]. Validation is limited by not knowing the true population size [26] because a complete census is rarely feasible. Instead, researchers tend to use additional methods for comparison, which fall into two categories. First, mark-recapture/resight methods have the potential to produce the most precise estimates of detection probability because a known number of marked individuals exists in the population and detection probability can be calculated as the proportion of marked individuals that are observed on subsequent surveys [3,26]. This method assumes that marked individuals behave similarly to unmarked individuals [85] and requires substantial effort in capture and marking, which varies among taxa. Second, non-marking methods that have higher detection probability, such as thermal imaging [28,31] or aerial surveys [24,35], set a lower bound for the proportion of animals missed by lower efficacy techniques. These methods require additional equipment and expertise to conduct and still estimate abundance given imperfect detection.

We opted to temporally replicate surveys to test the repeatability of counts and assess the stability of detection probability and animal availability during an annual monitoring period (similar to [24]). Two main benefits exist using temporal replication. First, no additional training or equipment is needed. Second, this method produces repeated measures of closed populations enabling better tests of whether observation-level covariates affect detection probability or availability. This is not possible using thermal imaging on the same night because no repeated measures exist. Distance sampling enables tests of observation-level covariates on detection probability via the detection function but cannot separate variation in animal availability from abundance without repeated measures. Researchers should choose the method that is best suited to address the largest suspected sources of bias in their system. In reality, a combination of validation techniques would likely continue to provide bias correction and confidence in population estimates. Such investment is probably most beneficial to long-term monitoring efforts whereby the information obtained can be applied to future protocol, data analysis, and interpretation.

Management Implications

Wildlife surveys are widely used to estimate animal abundance, despite well validated concerns that unexplained variation in detection can reduce the usefulness of survey counts. We show high consistency in survey counts among temporal replicates during a time period when animals were maximally exposed and available to sample. This high repeatability suggests that, given appropriate timing and ecological conditions, surveys can provide reliable population estimates. In particular, our results suggest deer managers should sample early in the spring green-up progression to maximize the proportion of the population that is available to sample. Systems

that can validate high repeatability among replicate counts can reduce annual monitoring effort to one survey per site per year (the norm for management agencies) while maintaining confidence in population estimates. Lastly, we show how temporal replication can be a cost-effective technique to evaluate the reliability of wildlife surveys, thereby providing a more effective basis for making informed management decisions.

Acknowledgements

We thank all the staff and volunteers that conducted spotlight surveys. We thank Bob Klaver and Chris Ensminger for discussion that improved the paper.

References

1. Callaghan CT, Santini L, Spake R, Bowler DE. Population abundance estimates in conservation and biodiversity research. Trends Ecol Evol 2024;39: 515–523.
2. Otis DL, Burnham KP, White GC, Anderson DR. Statistical inference from capture data on closed animal population. Wildl Monogr 1978;62: 3–135.
3. Seber GAF. A review of estimating animal abundance. Biometrics 1986;42: 267–292.
4. Schwarz CJ, Seber GAF. Estimating animal abundance: Review III. Stat Sci 1999;14: 427–456.
5. Kéry M, Royle JA. Applied Hierarchical Modeling in Ecology: Analysis of distribution, abundance and species richness in R and BUGS. Vol 1: Prelude and Static Models. Elsevier; 2016.
6. Kéry M, Royle JA. Applied Hierarchical Modeling in Ecology: Analysis of distribution, abundance and species richness in R and BUGS. Vol 2: Dynamic and Advanced Models. Elsevier; 2021.
7. Royle JA. *N*-mixture models for estimating population size from spatially replicated counts. Biometrics 2004;60: 108–115.
8. Madsen L, Royle JA. A review of N-mixture models. WIREs Comp Stat 2023;15: e1625.
9. Burnham KP, Anderson DR, Laake JL. Estimation of density from line transect sampling of biological populations. Ecol Monogr 1980;72: 1–2002.
10. Buckland ST, Anderson DR, Burnham KP, Laake JL, Borchers DL, Thomas L. Advanced Distance Sampling: Estimating Abundance of Biological Populations. Oxford University Press; 2004.

11. Miller DL, Rexstad E, Thomas L, Marshall L, Laake JL. 2019. Distance sampling in R. J Stat Softw 89:1–28.
12. Bishir J, Lancia RA. On catch-effort methods of estimating animal abundance. Biometrics 1996;52: 1457–1466.
13. Veech JA, Ott JR, Troy JR. Intrinsic heterogeneity in detection probability and its effect on N-mixture models. Methods Ecol Evol 2016;7: 1019–1028.
14. Duarte A, Adams MJ, Peterson JT. Fitting N-mixture models to count data with unmodeled heterogeneity: Bias, diagnostics, and alternative approaches. Ecol Mod 2018;374: 51–59.
15. Link WA, Schofield MR, Barker RJ, Sauer JR. On the robustness of N-mixture models. Ecology 2018;99: 1547–1551.
16. Latif QS, Valente JJ, Johnston A, Davis KL, Fogarty FA, Green AW, et al. Designing count-based studies in a world of hierarchical models. J Wildl Manage 2024; e22622.
17. Marsh H, Sinclair DF. Correcting for visibility bias in strip transect aerial surveys of aquatic fauna. J Wildl Manage 1989;53: 1017–1024.
18. Pollock KH, Marsh H, Bailey LL, Farnsworth GL, Simons TR, Alldredge MW. Separating components of detection probability in abundance estimation: an overview with diverse examples. In: Thompson WL. Sampling Rare or Elusive Species: Concepts, Designs, and Techniques for Estimating Population Parameters. Island Press; 2004. pp. 43–58.
19. Kéry M, Schmidt BR. Imperfect detection and its consequences for monitoring for conservation. Community Ecol 2008;9: 207–2008.
20. Robbins CS. 1981. Effect of time of day on bird activity. Stud Avian Biol 6:275–286.

21. Nykänen M, Jessopp M, Doyle TK, Harman LA, Cañadas A, Breen P, et al. Using tagging data and aerial surveys to incorporate availability bias in the abundance estimation of blue sharks (*Prionace glauca*). PLOS One 2018; doi.org/10.1371/journal.pone.0203122

22. Nichols JD, Hines JE, Sauer JR, Fallon FW, Fallon JE, Heglund PJ. A double-observer approach for estimating detection probability and abundance from point counts. Auk 2000;117: 393–408.

23. Anderson AS, Marques TA, Shoo LP, Williams SE. Detectability in audio-visual surveys of tropical rainforest birds: the influence of species, weather and habitat characteristics. PLoS ONE 2015;10: e0128464.

24. Blum J, Foley AM, DeYoung RW, Hewitt DG, Baumgardt J, Hellickson MW et al. Evaluation of drone surveys for ungulates in southwestern rangelands. Wildl Soc Bull 2024;48: e1515.

25. Garel M, Bonenfant C, Hamann JL, Klein F, Gaillard JM. Are abundance indices derived from spotlight counts reliable to monitor red deer *Cervus elaphus* populations? Wildl Biol 2010;16: 77–84.

26. Laake JL, Collier BA. Understanding implications of detection heterogeneity in wildlife abundance estimation. J Wildl Manage 2023;88: e22516.

27. McCullough DR. Evaluation of night spotlighting as a deer study technique. J Wildl Manage 1982;46: 963–973.

28. Focardi S, De Marinis AM, Rizzotto M, Pucci A. Comparative evaluation of thermal infrared imaging and spotlighting to survey wildlife. Wildl Soc Bull 2001;29: 133–139.

29. Gehrt SD. Evaluation of spotlight and road-kill surveys as indicators of local raccoon abundance. Wildl Soc Bull 2002;30: 449–456.

30. Scott DM, Waite S, Maddox TM, Freer RA, Dunstone N. The validity and precision of spotlighting for surveying desert mammal communities. J Arid Environ 2005;61: 589–601.

31. Collier BA, Ditchkoff SS, Raglin JB, Smith JM. Detection probability and sources of variation in white-tailed deer spotlight surveys. J Wildl Manage 2007;71: 277–281.

32. Collier BA, McCleery RA, Calhoun KW, Roques KG, Monadjem A. Detection probabilities of ungulates in the eastern Swaziland lowveld. South African J Wildl Res 2011;41: 61–67.

33. Strickland BA, Vilella FJ, Flynt RD. Long-term spotlight surveys of American alligators in Mississippi, USA. Herpetol Conserv and Biol 2018;13: 331–340.

34. Collier BA, Ditchkoff SS, Ruth Jr CR, Raglin JB. Spotlight surveys for white-tailed deer: Monitoring panacea or exercise in futility? J Wildl Manage 2013;77: 165–171.

35. Hodgson JC, Baylis SM, Mott R, Herrod A, Clarke RH. Precision wildlife monitoring using unmanned aerial vehicles. Sci Reports 2016;6: 22574.

36. Cypher BL. A technique to improve spotlight observations of deer. Wildl Soc Bull 1991;19: 391–393.

37. Whipple JD, Rollins D, Schacht WH. A field simulation for assessing accuracy of spotlight deer surveys. Wildl Soc Bull 1994;22: 667–673.

38. Gaidet-drapier N, Fritz H, Bourgarel M, Poilecot P, Chardonnet P, Coid C et al. Cost and efficiency of large mammal census techniques: comparison of methods for a participatory approach in a communal area, Zimbabwe. Biodivers Conserv 2006;15: 735–754.

39. Corlatti L, Gugiatti A, Pedrotti L. Spring spotlight counts provide reliable indices to track changes in population size of mountain-dwelling red deer *Cervus elaphus*. Wildl Biol 2016;22: 268–276.

40. Kaminski DJ, Harms TM, Coffey JM. Using spotlight observations to predict resource selection and abundance for white-tailed deer. J Wildl Manage 2019;83: 1565–1580.

41. Dinsmore S, Delaney D, Tyndall J. Density, population trends, and economic impacts of white-tailed deer in Iowa. A report in response to Iowa Senate File 581. 2024. Available from: www.legis.iowa.gov/docs/publications/DF/1462727.pdf

42. Amos M, Baxter G, Finch N, Lisle A, Murray P. I just want to count them! Considerations when choosing a deer population monitoring method. Wildl Biol 2014;20: 362–370.

43. Forsyth DM, Comte S, Davis NE, Bengsen AJ, Côté SD, Hewitt DG et al. Methodology matters when estimating deer abundance: a global systematic review and recommendations for improvements. J Wildl Manage 2022;86: e22207

44. Kaminski DJ, Harms TM, Evelsizer VD, Elliott JR. Iowa Spring Spotlight Survey: 2024 Summary. Iowa Department of Natural Resources 2023. Available from: www.iowadnr.gov/Portals/idnr/uploads/Hunting/spring_spotlight/Spotlight%20Survey%20Report_2024.pdf.

45. Rybarczyk WB. Evaluation of a spotlight survey technique as an index to Iowa white-tailed deer (*Odocoileus virginianus*) and raccoon (*Procyon lotor*) populations. Thesis. Iowa State University.

46. Montgomery GG. Nocturnal movements and activity rhythms of white-tailed deer. J Wildl Manage 1963;27: 422–427.

47. Zagata MD, Haugen AO. White-tailed deer movement at Pilot Knob State Park, Iowa. Proc Iowa Acad Sci 1974;81: Art. 11

48. Ozoga JT, Verme LJ. Winter feeding patterns of penned white-tailed deer. J Wildl Manage 1970;34: 431–439.

49. Beier P, McCullough DR. Factors influencing white-tailed deer activity patterns and habitat use. Wildl Monogr 1990;109: 1–51.

50. Hiller TL, Campa III H, Winterstein SR. Estimation and implications of space use for white-tailed deer management in southern Michigan. J Wildl Manage 2009;73: 201–209.

51. Hawkins RE, Klimstra WD. A preliminary study of the social organization of white-tailed deer. J Wildl Manage 1970;34: 407–419.

52. Dewitz J. National Land Cover Database (NLCD) 2019 Products [Data set]. U.S. Geological Survey. 2021. Available from: https://doi.org/10.5066/P9KZCM54

53. Elliott JR, Harms TM. Weather conditions affecting white-tailed deer harvest in Iowa. J Wildl Manage 2023;87: e22458.

54. US Department of Agriculture, Natural Resources Conservation Service, National Cartography & Geospatial Center. National Elevation Dataset 30 Meter 1-degree Tiles. 2001; Accessed 27 March 2023. Available from: https://www.usgs.gov/tools/download-data-maps-national-maphttps://www.usgs.gov/programs/national-geospatial-program/national-map.

55. Schloerke B, Cook D, Larmarange J, Briatte F, Marbach M, Thoen E et al. GGally: Extension to 'ggplot2'. R package version 2.2.1 2024. Available from: https://github.com/ggobi/ggally, https://ggobi.github.io/ggally/

56. Nakagawa S, Schielzeth H. A general and simple method for obtaining R^2 from generalized linear mix-effects models. Methods Ecol Evol 2013;4: 133–142.

57. Nakagawa S, Schielzeth H. Repeatability for Gaussian and non-Gaussian data: a practical guide for biologists. Biol Rev 2010;85: 935–956.

58. Kellner KF, Smith AD, Royle JA, Kéry M, Belant JL, Chandler RB. The *unmarked* R package: Twelve years of advances in occurrence and abundance modelling in ecology. Methods Ecol Evol 2023;14: 1408–1415.

59. Nichols JD, Thomas, L, Conn PB. Inferences about landbird abundance from count data: Recent advances and future directions. In Modeling Demographic Processes in Marked Populations. Springer; 2009. pp. 201–235.

60. Chandler RB, Royle JA, King DI. Inference about density and temporary emigration in unmarked populations. Ecology 2011; 92(7), 1429–1435.

61. Bartoń K. Multi-model inference. Package 'MuMIn'. 2009

62. Akaike H. A new look at the statistical model identification. IEEE Trans Automatic Control 1974;19: 716–723.

63. Burnham KP, Anderson DR. Model selection and multimodel inference: a practical information-theoretic approach, 2nd ed. Springer; 2002.

64. Arnold TW. Uninformative parameters and model selection using Akaike's Information Criterion. J Wildl Manage 2010;74: 1175–1178.

65. Vermote E. NOAA CDR Program. NOAA Climate Data Record (CDR) of VIIRS Normalized Difference Vegetation Index (NDVI), Version 1. NOAA National Centers for Environmental Information. 2022. Available from: https://doi.org/10.25921/gakh-st76

66. Merkle JA, Monteith KL, Aikens EO, Hayes MM, Hersey KR, Middleton AD et al. Large herbivores surf waves of green-up during spring. Proc Roy Soc B 2016;283: 20160456. http://dx.doi.org/10.1098/rspb.2016.0456

67. Hirth DH. Social behavior of white-tailed deer in relation to habitat. Wildl Monogr 1977;53: 3–55.

68. Lagory KE. Habitat, group size, and the behavior of white-tailed deer. Behaviour 1986;98: 168–179.

69. Sorensen VA, Taylor DH. The effect of seasonal change on the group size, group composition, and activity budget of white-tailed deer, Odocoileus virginianus. Ohio J Sci 1995;95: 321–324.

70. Creel S, Schuette P, Christianson D. Effects of predation risk on group size, vigilance, and foraging behavior in an African ungulate community. Behav Ecol 2014;25: 773–784.

71. Takada H, Minami M. Open habitats promote female group formation in a solitary ungulate: the Japanese serow. Behav Ecol Sociobiol 2021;75: 60.

72. McGovern PG, Dinsmore SJ, Blanchong JA. Survival of white-tailed deer fawns in central Iowa. PLOS One 2020; https://doi.org/10.1371/journal.pone.0229242

73. Aben J, Pellikka P, Travis JMJ. A call for viewshed ecology: Advancing our understanding of the ecology of information through viewshed analysis. Methods Ecol Evol 2018;9: 624–633.

74. Curtis KM, Jennings MK, Laake J, Botta R, Colby J, Tomaszewski EM et al. Hybrid mark-resight approaches account for heterogeneous detection bias in aerial surveys. Ecol Solutions Evidence 2023; 4:e12289.

75. Murphy DA. Deer range appraisal in the Midwest. In White-tailed Deer in the Midwest: A Symposium. USDA Forest Service Research Paper 1970; 39:2–10.

76. Barker RJ, Schofield MR, Link WA, Sauer JR. On the reliability of N-mixture models for count data. Biometrics 2018;74: 369–377.

77. Ficetola GF, Barzaghi B, Melotto A, Muraro M, Lunghi E, Canedoli C et al. Sci Rep 2018;8: 10357.

78. Costa A, Romano A, Salvidio S. Reliability of multinomial N-mixture models for estimating abundance of small terrestrial vertebrates. Biodivers Conserv 2020;29: 2951–2965.

79. Buckland ST, Anderson DR, Burnham KP, Laake JL, Borchers DL, Thomas L. Introduction to Distance Sampling: Estimating Abundance of Biological Populations. Oxford University Press; 2001.

80. O'Connell Jr AF, Talancy NW, Bailey LL, Sauer JR, Cook R, Gilbert AT. Estimating site occupancy and detection probability parameters for meso- and large mammals in a coastal ecosystem. J Wildl Manage 2006;70: 1625–1633.

81. Couturier T, Cheylan M, Bertolero A, Astruc G, Besnard A. Estimating abundance and population trends when detection is low and highly variable: a comparison of three methods for the Hermann's Tortoise. J Wildl Manage 2013;77: 454–462.

82. Marques TA, Buckland ST, Borchers DL, Tosh D, McDonald RA. Point transect sampling along linear features. Biometrics 2010;66: 1247–1255.

83. Beaver JT, Harper CA, Kissell Jr RE, Muller LI, Basinger PS, Goode MJ et al. Aerial vertical-looking infrared imagery to evaluate bias of distance sampling techniques for white-tailed deer. Wildl Soc Bull 2006;38: 419–427.

84. Brack IV, Kindel A, de Oliveira LFB, Lahoz-Monfort JJ. Optimally designing drone-based surveys for wildlife abundance estimation with N-mixture models. Methods Ecol Evol 2023;14: 898–910.

85. Nichols JD, Hines JE, Pollock KH. Effects of permanent trap response in capture probability on Jolly-Seber capture-recapture model estimates. J Wildl Manage 1984;48: 289–294.

Fig 1

Click here to access/download;Figure;Fig 1.jpg

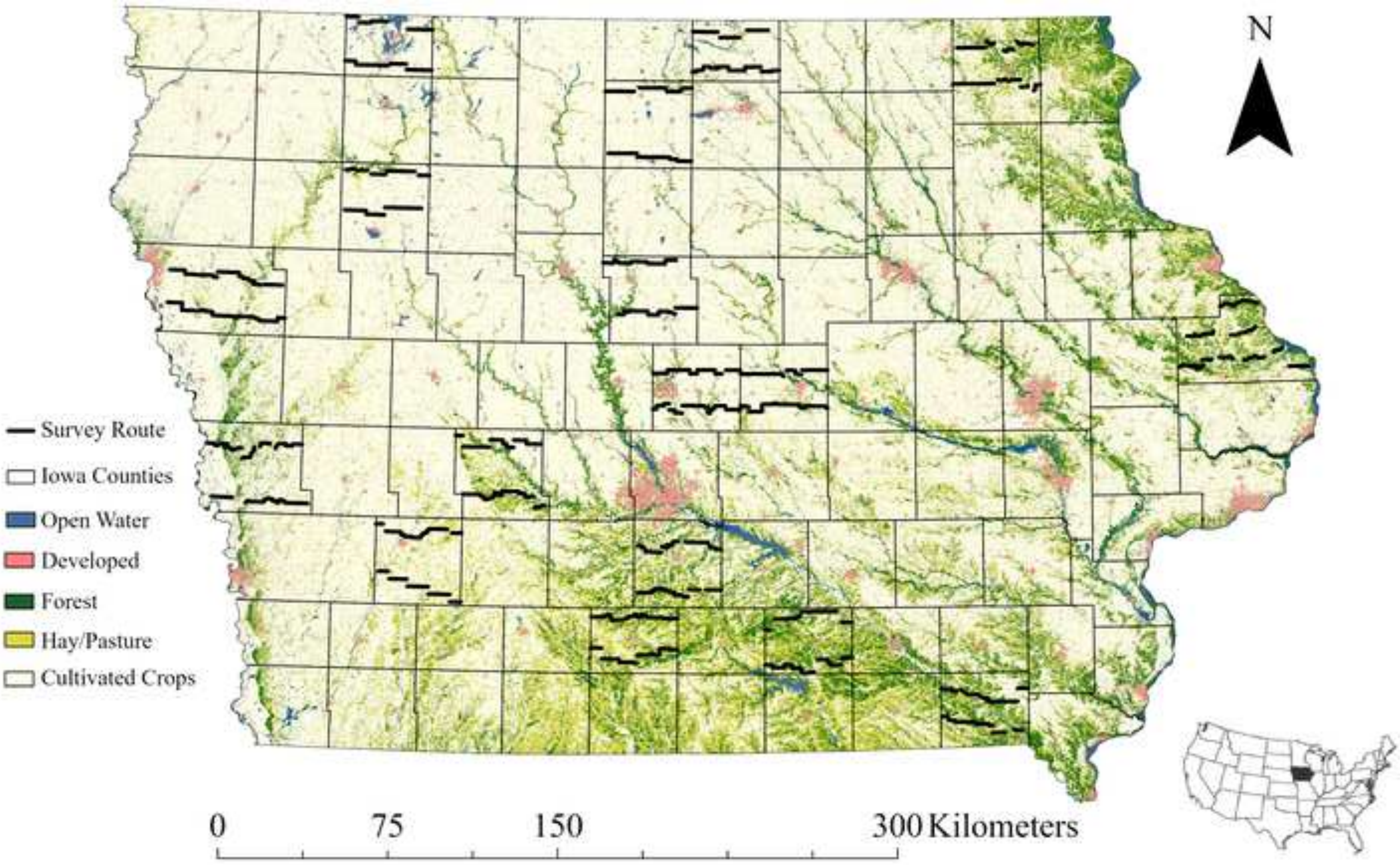

Fig 2

Click here to access/download;Figure;Fig 2.pdf

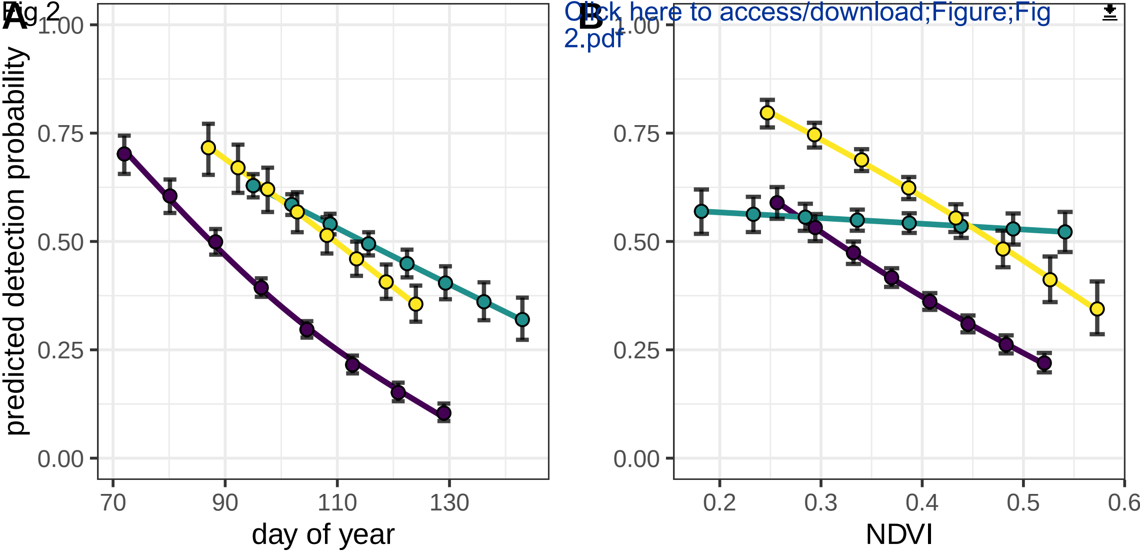

Fig 3

Click here to access/download;Fi

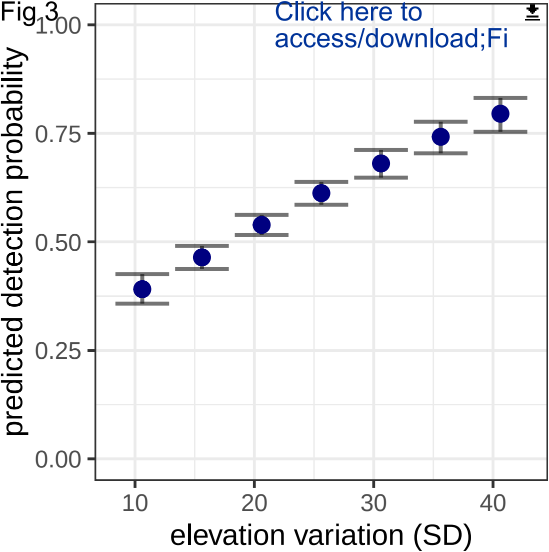

Fig 4

Click here to access/download;Figure;Fig 4.pdf

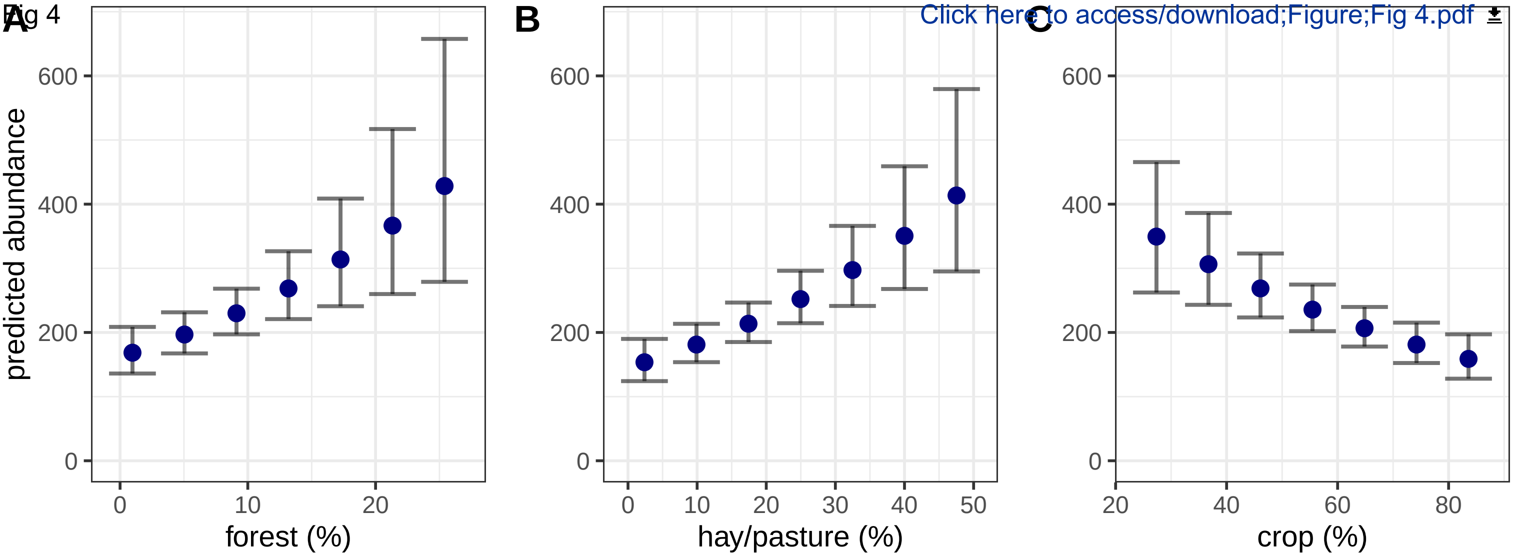

Click here to access/download
Supporting Information
Supplementary Material.docx

Click here to access/download
Supporting Information
archived_data.xlsx
