# Supplementary material for: Repeatability of wildlife surveys for estimating abundance: A method to assess the consistency of detection probability and animal availability
Source: PLoS One. 2025 Apr 23;20(4):e0321619. doi: 10.1371/journal.pone.0321619 (PMC12017516; doi:10.1371/journal.pone.0321619)
Supplement: Attachment — Submitted filename: Response to Reviewers.docx [file pone.0321619.s017.docx]

Dear Dr. Rich,

Thank you for the reviews of our manuscript. We appreciate the time and effort that you and the reviewers put forth to improve the paper. We found all the reviewer’s comments helpful and we have now addressed each concern raised. Our responses are outlined below (in bold) immediately following each comment. Line numbers reference the marked version of the manuscript.

Sincerely,

David Delaney

Journal requirements

When submitting your revision, we need you to address these additional requirements.

1. Please ensure that your manuscript meets PLOS ONE's style requirements, including those for file naming. The PLOS ONE style templates can be found at

<https://journals.plos.org/plosone/s/file?id=wjVg/PLOSOne_formatting_sample_main_body.pdf> and

<https://journals.plos.org/plosone/s/file?id=ba62/PLOSOne_formatting_sample_title_authors_affiliations.pdf>

**We have edited to match PLOS ONE’s style requirements.**

2. In your Methods section, please provide additional information regarding the permits you obtained for the work. Please ensure you have included the full name of the authority that approved the field site access and, if no permits were required, a brief statement explaining why.

**Lines 149-152: We added the following section:**

**“Permits**

**We collected data by observing animals from publicly owned roads, and animals were not captured or marked. Therefore, our study did not require field site permission or Institutional Animal Care and Use Committee approval.”**

3. We note that Figure 1 in your submission contain [map/satellite] images which may be copyrighted. All PLOS content is published under the Creative Commons Attribution License (CC BY 4.0), which means that the manuscript, images, and Supporting Information files will be freely available online, and any third party is permitted to access, download, copy, distribute, and use these materials in any way, even commercially, with proper attribution. For these reasons, we cannot publish previously copyrighted maps or satellite images created using proprietary data, such as Google software (Google Maps, Street View, and Earth). For more information, see our copyright guidelines: <http://journals.plos.org/plosone/s/licenses-and-copyright>.

We require you to either (1) present written permission from the copyright holder to publish these figures specifically under the CC BY 4.0 license, or (2) remove the figures from your submission:

**This figure does not directly display satellite images. We created this figure from USGS NLCD data. We now cite the source of the base map data.**

**Line 126: We now specify within the figure caption: “Base map data provided by [54].”**

4. We note that there is identifying data in the Supporting Information file < archived_data.xlsx>. Due to the inclusion of these potentially identifying data, we have removed this file from your file inventory. Prior to sharing human research participant data, authors should consult with an ethics committee to ensure data are shared in accordance with participant consent and all applicable local laws.

**We are unaware of personally identifying information in our dataset. Perhaps county names were perceived as the names of people. Nevertheless, we have reviewed and uploaded our archived dataset.**

5. Please include captions for your Supporting Information files at the end of your manuscript, and update any in-text citations to match accordingly. Please see our Supporting Information guidelines for more information: <http://journals.plos.org/plosone/s/supporting-information>.

**We now include captions for our Supporting Information files at the end of the manuscript.**

**We have also updated in-text citations.**

6. Please review your reference list to ensure that it is complete and correct. If you have cited papers that have been retracted, please include the rationale for doing so in the manuscript text, or remove these references and replace them with relevant current references. Any changes to the reference list should be mentioned in the rebuttal letter that accompanies your revised manuscript. If you need to cite a retracted article, indicate the article’s retracted status in the References list and also include a citation and full reference for the retraction notice.
**We have reviewed and updated our reference list.**


***Reviewer #1*:**

1. I think the title undersells both the objectives of the paper and what you did, you looked at more than just repeatability.

**We appreciate this point. We hope our method will be implemented in study systems other than deer in Iowa so we refrained from putting system-specific information in the title.**

**We expanded our title to the following: “Repeatability of wildlife surveys for estimating abundance: A method to assess the consistency of detection probability and animal availability”**

2. Line 62-“For systems with repeatable counts and predictable detection probability, researchers can reduce visits to one sample per site and reallocate effort elsewhere.” Are you assuming then that if the count is repeatable that availability bias is low? In the Introduction you very clearly lay out the 2 main sources of bias to a count method and mention that it is hard to identify the two, but I do not recall any further specific discussion of availability after that. I think that in your suggestion that sightability is very high in your open system you are assuming that availability is high? But you also suggest that viewing distance changes with elevation variation, could that affect availability?
**We posit that highly repeatable counts indicate consistent detection and availability, such that counts do accurately index population size.**

**Lines 359 & 361: In the Discussion, under ‘Comparison with other systems’,** **we added text to note that our discussion of detection probability also includes animal availability.**

3. Why does topographic variation positively affect detection? In theory if you include a variable in a model and it generates fit opposite to expectation then you should drop that variable from the model because the fit is spurious because you cannot explain it. Are you sure that the variation in elevation is not positively affecting abundance and that you are mistaking one factor for the other? Maybe there is more food in places with topographic variation?

**This is the second analysis of our roadside surveys in Iowa that found a positive effect of elevation variation on detection probability. In the other paper, we show that sites that have reduced viewsheds (because of hilly, forested habitat) have higher detection probability. This is because these sites have more visible area close to the transect, where detection is high, compared to flat sites that have more visible area far from the transect, where detection is lower. This paper has also received a revise and resubmit decision elsewhere and is not yet citable. Nevertheless, this adds support that this finding is real and not spurious.**

**Lines 325-338: We elaborate on this in the Discussion: “Elevation variation was the only site-level covariate to have a considerable effect on detection probability. Percent forest, hay/pasture, and crops had significant associations with detection probability, but these may result from percent landcover correlations with elevation variation (Fig S5). We hypothesized that elevation variation could influence detection probability by increasing the visual complexity of the landscape and increasing the difficulty for an observer to detect deer. In contrast, we observed higher detection probability at sites with greater elevation variation. Sites with greater elevation variation likely have smaller viewsheds, which reduces the area that is sampleable (*sensu* [37,75]). Because elevation variation is likely to shorten the viewshed closer to the transect where visibility is greater, average detection probability over the area that can be sampled should be higher. Smaller viewsheds may also reduce observer fatigue [76] and focus observers on areas where deer could be detected. Topographically diverse sites have also produced reliable spotlight survey estimates of red deer in mountainous regions of Italy [39] and France [25], and aerial surveys of mule deer (*Odocoileus hemionus*) found higher detection in areas with increased ruggedness [76].”**

**See also response to point 4 below.**

4. In the Introduction or Methods section please add a sentence explaining to the reader how a mixture model can estimate both abundance and detection from simple count data. This is not an intuitive idea but is important to your paper and especially important to my question above about identifiability.
**Lines 178-183: We added the following sentences: “N-mixture models estimate detection probability by assessing the variation in counts among temporally replicated surveys and estimate abundance based jointly on the variation in counts among sites and average detection probability. The effect of a single predictor can be simultaneously estimated on both detection and abundance because the N-mixture model derives abundance and detection probability effects from these different sources of variation in counts [60].”**

5. Are there large predators in your system that may affect nocturnal choice of habitat by deer, and therefore availability?
**Coyotes are the largest non-human predators in our system, which are not predators of healthy adult deer in open habitat.**

6. In line 278 can you conclude that temporal variation is only partially related to green-up and equally influenced by other factors? If so, then I suggest you add a closing sentence stating that clearly. This is important for future sampling design.
**With the data on hand, we cannot conclude that temporal variation is only partially related green-up.**

**Lines 302-306: We added the following text for additional context: “One reason could be that cloud cover blocked parts of satellite images that we used to extract NDVI such that this measure did not perfectly reflect vegetative green-up throughout our entire dataset. Although we developed a data processing framework to reduce cloud cover bias (S1 Appendix), cloud cover in images from 2018 likely explains the shallow slope in Fig 2B.”**

7. Mgmt Recommendations: here can you not describe or suggest the methodological improvements you suggest should be applied to these surveys? What is the preferred survey window? What covariates should be collected, which can be dropped? Which model should the practitioners use to index or estimate deer abundance. Can you not now estimate abundance using the mixture model and avoid the index all together? Can they apply the model to the historical data and expect better retrospective inference?
**We have retitled this section to, ‘Conclusions’, which better fits what we hope readers take away from this study. We are cautious to suggest researchers should take our findings and adjust their study designs or models. Instead we hope they consider implementing temporal replication to assess the consistency of detection and availability to inform confidence in their survey counts.**

**Lines 429-431: We do have one recommendation in this paragraph which we feel is broadly helpful for deer research: “In particular, our results suggest deer managers should sample early in the spring green-up progression (or before) to maximize the proportion of the population that is available to sample.”**

8. I am thinking about the paper from a statistical point of view because I cannot help but think you learned more than you have presented in your Mgmt Implications section. I am rambling a bit below while I try to put my finger on what you have overlooked.

Step 1 examined precision and you were quite lucky in that it is high. What would you have recommended if it was low? Given it is high then can you word your recommendation in lines 382-385 more directly-only one count is needed if it is done between these dates?
**Lines 101-104: “In contrast, poor consistency in counts among temporal replicates that was not explained by covariates would suggest spotlight surveys do not produce meaningful counts during this period and alternative sampling techniques should be explored.”**

**As noted above, we prefer to keep this Conclusion section about the application of the method and less about the specifics of our system.**

Step 2 examined detection success and given precision was high this should be the main signal in the data? Or can you say, based on the strength of your abundance model how much abundance may have confounded detection? Here your hypothesis that seasonal timing effects detection was supported while the green-up hypothesis was less supported. Again, given the strength of those results, I think you can make a recommendation about actual survey timing in your system, and, for the benefit of others, tell use about the typical weather and deer social behaviour during that time. Can you say why the seasonal effect varied among years or, is that likely just due to variation in the data among years (ie random)? As above, is the positive association with pastureland identifiable as a detection effect, or could it be confounded with an influence on abundance? Regardless of your response, should this relationship not be mentioned in the Mgmt Implications section? My thought is to leave the habitat variable out of the detection model because you already know it influences abundance.
**See response to point 7 above. We elaborate on the system-specific implications within the main text of the Discussion (i.e., prior to the Conclusion paragraph).**

**Lines 178-183: We added the following sentences on potential confounding: “N-mixture models estimate detection probability by assessing the variation in counts among temporally replicated surveys and estimate abundance based jointly on the variation in counts among sites and average detection probability. The effect of a single predictor can be simultaneously estimated on both detection and abundance because the N-mixture model derives abundance and detection probability effects from these different sources of variation in counts [60].”**

Step 3 examined influences on abundance and here availability may be the largest influence because precision is high, and you have accounted for a number of factors influencing detection probability. As I mentioned above, it seems to me you now have the tools to estimate abundance and again, whether you agree with me or not, I think you need to mention in the Mgmt Implications section that you examined abundance, while controlling for precision (sort of), and detection, and found abundance was related to habitat type. Which is something I suspect your surveyors have been seeing for years.
**See response to point 7 above. We elaborate on the system-specific implications within the Abundance section of the Discussion (i.e., prior to the Conclusion paragraph).**

**Lines 29-31: We added the following sentence to the Abstract: “****Deer abundance was greater at sites with higher percent landcovers of forest and hay/pasture and was lower at sites with higher landcover in crops.”**

***Reviewer #2*:**

This is a clear and concise paper on the repeatability of count data and methods for assessing it. I have minor line comments, but the more major of those are asking why the mixed model was fit using a Gaussian distribution and a recommendation to rewrite the N-mixture model section because it was difficult to follow which covariates were used when and why.


26-28: Not sure what this means. If you’re doing single-visit sampling how can you use temporal replication to assess them?

**Lines 34-37: We edited the following sentence: “Finally, we show that temporal replication of surveys is a logistically feasible method to assess the reliability of abundance estimates from study designs that are normally conducted with single visits.**

141-143: What is the GGally package actually calculating? Why couldn’t you just look at the correlation coefficient?

**The *GGally* package calculates the correlation coefficient, which could have been calculated without the use of *GGally*. We opted to cite this package because of it enabled efficient exploration of univariate and bivariate distributions with publication-quality plots and bivariate correlation coefficients.**

145: Does this mean every site-year combination was treated as a random effect?

**Lines 159-161: We added text to the following sentence: “****To quantify the repeatability (*R*) of survey counts, we used linear mixed models in the package *rptR* [57] with site–year as a random effect (i.e., “site–year” was fit as a single term in our model, which fit a random intercept for each site–year combination in the dataset).**

146: Need to indicate if high values of this fraction indicate high or low repeatability.
**Lines 164-165: We have added the following sentence: “Repeatability ranges from 0 to 1, and a value of 1 would indicate that all variation is explained by site–year.”**

148: Why Gaussian and not Poisson?
**Lines 165-167: We added text to the following sentence: “****We modeled the number of deer observed during each survey as a response variable with a Gaussian error distribution, which was a better fit than a Poisson distribution (Δ Akaike information criterion (AIC) = 252).”**

154-155: If you are using a negative binomial here, no reason not to use Poisson/negative binomial in the mixed model.
**See prior response.**

161: “fit” instead of “run”
**Addressed as suggested.**

163: Define uninformative parameter
**Lines 196-198: We added text to the following sentence: “We determined the best model as the model with the lowest AIC [64,65] and did not consider models with uninformative parameters (i.e., predictors that did not have a significant relationship with the response) [66].”**

164-170: Is this for the N mixture model as well? Why not include NDVI originally?
**We did not want to fit day of year and NDVI in the same model because of their covariation. We also wanted to build comparable models that simply switched out day of year for NDVI for direct tests of their explanatory power on detection.**

171-181: Does this mean elevation either appeared in the model for abundance OR detection, but no model was fit with them together? Just having trouble following the sequence of models.
**As we noted above, variables can be simultaneously fit on abundance and detection without issue.**

**See next response for more detailed response.**

178-180: It seems like the fact that you included transect length as a covariate is information that should come earlier. I would recommend rewriting the N-mixture model section quite a bit, it’s hard to follow which covariates are appearing when and why. Covariates that are in all the models regardless should be introduced first, then the core set of covariates you are testing, then the additional covariates and which models they are going into.
**We added headers and elaborated on how and why we built N-mixture models sequentially.**

**“****N-mixture model framework**

**To test whether a variety of observation- and site-level covariates relate to detection and abundance, we built a series of N-mixture models in the unmarked package [59]. Because of covariation among potential predictors, limited sample size, and potential global model complexity, we built N-mixture models in four steps to identify final models: 1) identification of observation-level variables on detection, 2) comparison between day of year and NDVI on detection, 3) addition of site-level variables on detection, and 4) addition of site-level variables on abundance.**

**N-mixture models estimate detection probability by assessing the variation in counts among temporally replicated surveys and estimate abundance based jointly on the variation in counts among sites and average detection probability. The effect of a single predictor can be simultaneously estimated on both detection and abundance because the N-mixture model derives abundance and detection probability effects from these different sources of variation in counts [60]. As is the case for most N-mixture models [19,61,62], we were not able to separate detection from availability. We modeled abundance with a negative binomial distribution (c-hat = 1.58) because Poisson (Δ AIC = 2778) and zero-inflated Poisson (Δ AIC = 2780) yielded poorer model fits for intercepts-only models in an a priori analysis.**

**Observation-level variables on detection**

**We included temperature, humidity, day of year, and year (as a factor) as predictors of detection probability and fit an intercept on abundance. We also included an interaction of day of year and year because sampling began and concluded at different times each year in response to variation in vegetation green-up phenology among years. We used the dredge function in the package MuMIn [63] to fit all possible models that varied in the combination of predictors. We determined the best model as the model with the lowest AIC [64,65] and did not consider models with uninformative parameters** **(i.e., predictors that did not have a significant relationship with the response) [66].**

**Comparison between day of year and NDVI on detection**

**Based on results from the prior model, we also calculated an index of vegetation green-up (Normalized Difference Vegetation Index, NDVI,** **S1 Appendix). To do this, we used NDVI data available from the National Oceanic and Atmospheric Administration Climate Data Record [67], which was correlated with day of year (r = 0.30 in global dataset; r = 0.41–0.47 within year subsets, S1 Fig, S2 Fig, S3 Fig, and S4 Fig). We built two additional models to test how green-up compared with day of year in predicting detection probability: (1) a model with NDVI and year as predictors and (2) a model with an interaction of NDVI and year as predictors.**

**Addition of site-level variables on detection**

**To test whether site-level variables covaried with detection probability, we selected the prior top model and added the following predictors: elevation variation, forest percent, hay/pasture percent, and crop percent. Each additional predictor was fit in a separate model because of high correlation among these variables (S5 Fig).**

**Addition of site-level variables on abundance**

**To test whether site-level variables covaried with abundance, we selected the prior top model for detection probability and ran a series of models including the addition of elevation variation, forest percent, hay/pasture percent, and crop percent as predictors on abundance. Again, each of these predictors was run in separate models because of correlation among these variables. Each model of abundance included transect length as a covariate to account for variable sampling effort among sites. We considered the model with the lowest AIC that included covariates on both detection probability and abundance as our top model.”**

184: This R is not a correlation coefficient correct? It’s proportion of variance explained by site-year combination? Still having a hard time thinking about what a high value means here.
**Correct.**

**Lines 164-165: We have added the following sentence: “Repeatability ranges from 0 to 1, and a value of 1 would indicate that all variation is explained by site–year.”**

Discussion: Given you found that abundance and detection probability were influenced by the same covariates, do you have confounding in the two processes? Can you actually separate abundance from detection/availability?

**Lines 178-182: We added the following sentences: “N-mixture models estimate detection probability by assessing the variation in counts among temporally replicated surveys and estimate abundance based jointly on the variation in counts among sites and average detection probability. The effect of a single predictor can be simultaneously estimated on both detection and abundance because the N-mixture model derives abundance and detection probability effects from these different sources of variation in counts [60].”**
